# Animal Models of Ehlers–Danlos Syndromes: Phenotype, Pathogenesis, and Translational Potential

**DOI:** 10.3389/fgene.2021.726474

**Published:** 2021-10-12

**Authors:** Robin Vroman, Anne-Marie Malfait, Rachel E. Miller, Fransiska Malfait, Delfien Syx

**Affiliations:** ^1^Center for Medical Genetics, Department of Biomolecular Medicine, Ghent University, Ghent, Belgium; ^2^Division of Rheumatology, Rush University Medical Center, Chicago, IL, United States

**Keywords:** Ehlers–Danlos syndromes, EDS, animal models, mouse, zebrafish

## Abstract

The Ehlers–Danlos syndromes (EDS) are a group of heritable connective tissues disorders mainly characterized by skin hyperextensibility, joint hypermobility and generalized tissue fragility. Currently, 14 EDS subtypes each with particular phenotypic features are recognized and are caused by genetic defects in 20 different genes. All of these genes are involved in the biosynthesis and/or fibrillogenesis of collagens at some level. Although great progress has been made in elucidating the molecular basis of different EDS subtypes, the pathogenic mechanisms underlying the observed phenotypes remain poorly understood, and consequentially, adequate treatment and management options for these conditions remain scarce. To date, several animal models, mainly mice and zebrafish, have been described with defects in 14 of the 20 hitherto known EDS-associated genes. These models have been instrumental in discerning the functions and roles of the corresponding proteins during development, maturation and repair and in portraying their roles during collagen biosynthesis and/or fibrillogenesis, for some even before their contribution to an EDS phenotype was elucidated. Additionally, extensive phenotypical characterization of these models has shown that they largely phenocopy their human counterparts, with recapitulation of several clinical hallmarks of the corresponding EDS subtype, including dermatological, cardiovascular, musculoskeletal and ocular features, as well as biomechanical and ultrastructural similarities in tissues. In this narrative review, we provide a comprehensive overview of animal models manifesting phenotypes that mimic EDS with a focus on engineered mouse and zebrafish models, and their relevance in past and future EDS research. Additionally, we briefly discuss domestic animals with naturally occurring EDS phenotypes. Collectively, these animal models have only started to reveal glimpses into the pathophysiological aspects associated with EDS and will undoubtably continue to play critical roles in EDS research due to their tremendous potential for pinpointing (common) signaling pathways, unveiling possible therapeutic targets and providing opportunities for preclinical therapeutic interventions.

## Introduction

The Ehlers–Danlos syndromes (EDS) comprise a group of rare heritable connective tissue disorders, clinically hallmarked by skin hyperextensibility and fragility, generalized joint hypermobility, abnormal wound healing, easy bruising, and widespread connective tissue friability (Malfait et al., 2017). Additional clinical features differ among the EDS subtypes and include potentially life-threatening cardiovascular complications, fragility of hollow organs, involvement of the musculoskeletal and/or ocular system as well as chronic, widespread pain, which can all contribute to severe disability, compromising patients’ quality of life, and/or early mortality (Malfait et al., 2020).

Originally suspected to be a disorder affecting the collagen “wickerwork” based on electron microscopy studies (Jansen, 1955), early biochemical and genetic studies indeed identified defects in the primary structure of fibrillar collagens (types I, III, and V) or their modifying enzymes in human patients displaying different subtypes of EDS (Beighton et al., 1998). More recently, defects affecting other extracellular matrix (ECM) molecules (e.g., tenascin-X, enzymes involved in glycosaminoglycan biosynthesis of proteoglycans, etc.) further expanded the molecular and clinical heterogeneity of this syndrome. These findings laid the basis for the revised *International Classification of the Ehlers–Danlos Syndromes*, published in 2017, which provides a regrouping of EDS based on the underlying genetic defect and affected pathways (Malfait et al., 2017). To date, 14 distinct EDS subtypes are recognized, of which 13 have been molecularly elucidated and are caused by defects in 20 different genes (Malfait et al., 2017, 2020). All of these EDS-related defects compromise proper collagen biosynthesis, fibrillogenesis and/or supramolecular organization of collagen fibrils at some level as demonstrated by altered collagen fibril ultrastructure on transmission electron microscopy (TEM) in patients’ dermis (Hausser and Anton-Lamprecht, 1994; Proske et al., 2006). Although for many EDS types the scope of both genetic and allelic heterogeneity is well appreciated, the pathways linking the genetic changes to the phenotype remain largely elusive. An overview of EDS-related genes and proteins and their corresponding clinical presentation is provided in Table 1.

**TABLE 1:** Overview of the Ehlers-Danlos syndrome (EDS) types, causal gene and protein, inheritance pattern (IP) and the major and minor clinical criteria as defined by the 2017 International EDS Classification with the reported animal models indicated.

EDS subtype	Gene	Protein	IP	Major criteria	Minor criteria	Reported animal models
**a. Disorders of collagen primary structure and collagen processing**						
Classical (cEDS)	*COL5A1 COL5A2* (rare: *COL1A1* p.(Arg312Cys))	α1-chain of type V procollagen α2-chain of type V procollagen α1-chain of type I procollagen	AD	Skin hyperextensibility with atrophic scarring Generalized joint hypermobility	Easy bruising Soft doughy skin Skin fragility (or traumatic splitting) Molluscoid pseudotumors (bluish-grey, spongy nodules, which are herniations of subcutaneous fat, seen over easily traumatized areas) Subcutaneous spheroids Hernia (or history thereof) Epicanthal folds Complications of joint hypermobility (e.g., sprain, (sub)luxation, pain, flexible flatfoot) Family history of a 1^st^ degree relative who meets criteria	*Col5a1*^+/–^ *(Mm) Col5a2^*pN/pN*^ (Mm) Col5a2*^+/–^*(Mm) /*
Vascular (vEDS)	*COL3A1* (rare: *COL1A1* p.(Arg312Cys) p.(Arg574Cys) p.(Arg1093Cys))	α1-chain of type III procollagen α1-chain of type I procollagen	AD	Family history of vEDS with documented pathogenic variant in *COL3A1* Arterial rupture at young age Spontaneous sigmoid colon perforation in the absence of known colon disease Uterine rupture during 3^*rd*^ trimester of pregnancy Carotid-cavernous sinus fistula (in the absence of trauma)	Bruising unrelated to identified trauma and/or in unusual sites such as cheeks and back Thin, translucent skin with increased venous visibility Characteristic facial features: large eyes, periorbital pigmentation, small chin, sunken cheeks, thin nose and lips, lobeless ears Spontaneous pneumothorax Acrogeria Talipes equinovarus Congenital hip dislocation Small joint hypermobility Tendon and muscle rupture Gingival recession and gingival fragility Early-onset varicose veins	*Col3a1^*tm*1*Jae*^/J Col3a1^*m*1*Lsmi*/+^ Col3a1^*Tg*–*G*182*S*^ Col3a1^*G*183*R*/+^ Col3a1^*G*209*S/*+^ Col3a1^*G938D/*+^ (all Mm) /*
Arthrochalasia (aEDS)	*COL1A1 COL1A2*	α1-chain of type I procollagen α2-chain of type I procollagen	AD	Congenital bilateral hip dislocation Severe generalized joint hypermobility with multiple dislocations Skin hyperextensibility	Muscle hypotonia Kyphoscoliosis Radiologically mild osteopenia Tissue fragility, including atrophic scars Easy bruising	*/* /
Dermatosparaxis (dEDS)	*ADAMTS2*	A disintegrin and metalloproteinase with thrombospondin Motifs 2 (ADAMTS-2)	AR	Extreme skin fragility with congenital or postnatal tears Craniofacial features: large fontanel, puffy eyelids, excessive peri-orbital skin, downslanting palpebral fissures, blue sclerae, hypoplastic chin Progressively redundant, almost lax skin with excessive skin folds at wrists and ankles Increased palmar wrinkling Severe bruisability with risk of subcutaneous hematoma Umbilical hernia Postnatal growth retardation with short limbs Perinatal complications related to tissue fragility	Soft and doughy skin texture Skin hyperextensibility Atrophic scars Generalized joint hypermobility Complications of visceral fragility (e.g., rectal prolapse, bladder or diaphragm rupture) Delayed motor development Osteopenia Hirsutism Tooth abnormalities Refractive errors Strabismus	*Adamts2*^–^*^/^*^–^ *(Mm)*
Cardiac-valvular (cvEDS)	*COL1A2*	α2-chain of type I procollagen (total absence)	AR	Severe progressive cardiac-valvular insufficiency Skin involvement Joint hypermobility (generalized or restricted to small joints)	Inguinal hernia Pectus deformity Joint dislocations Foot deformities: pes planus, pes plano valgus, hallux valgus	*col1a2*^–^*^/^*^–^ *(Dr)*
**b. Disorders of collagen folding and collagen cross-linking**						
Kyphoscoliotic (kEDS)	*PLOD1 FKBP14*	Lysyl hydroxylase 1 (LH1) FK506 Binding Protein 22 KDa (FKBP22)	AR	Congenital muscle hypotonia Congenital or early onset kyphoscoliosis Generalized joint hypermobility with (sub)luxations	Skin hyperextensibility Easy bruising Rupture/aneurysm of medium-sized artery Osteopenia/osteoporosis Blue sclerae Umbilical or inguinal hernia Pectus deformity Marfanoid habitus Talipes equinovarus Refractive errors kEDS*-PLOD1:* Skin fragility Microcornea Characteristics craniofacial features kEDS*-FKBP14:* Congenital hearing impairment Muscle atrophy Bladder diverticula	*Plod1*^–^*^/^*^–^ *(Mm) /*
**c. Disorders of structure and function of the myomatrix**						
Classical-like (clEDS)	*TNXB*	Tenascin-X (TNX)	AR	Skin hyperextensibility with velvety skin texture and absence of atrophic scarring Generalized joint hypermobility Easy bruisable skin/spontaneous ecchymoses	Foot deformities Edema in legs in absence of cardiac failure Mild proximal and distal muscle weakness Axonal polyneuropathy Atrophy of muscle in hands and feet Acrogeric hands, mallet finger(s), clino- or brachydactyly Vaginal/uterine/rectal prolapse	*Tnxb*^–^*^/^*^–^ *(Mm)*
Myopathic (mEDS)	*COL12A1*	α1-chain of type XII procollagen	AD AR	Congenital muscle hypotonia and/or muscle atrophy Proximal joint contractures Hypermobility of distal joints	Soft, doughy skin Atrophic scarring Motor developmental delay Myopathy on muscle biopsy	*Col12a1*^–^*^/^*^–^ *(Mm)*
**d. Disorders of glycosaminoglycan biosynthesis**						
Musculocontractural (mcEDS)	*CHST14 DSE*	Dermatan 4-*O*-sulfotransferase-1 (D4ST1) Dermatan sulfate epimerase-1 (DS-epi1)	AR	Congenital multiple contractures: typically adduction/flexion contractures and talipes equinovarus Characteristic craniofacial features: large fontanelle, short downslanting palpebral fissures, blue sclerae, hypertelorism, short nose with hypoplastic columella, low-set and rotated ears, long philtrum with thin upper lip vermillion, small mouth and hypoplastic chin	Recurrent/chronic dislocations Pectus deformities Spinal deformities Peculiar fingers Progressive talipes deformities Large subcutaneous hematomas Chronic constipation	*Chst14*^–^*^/^*^–^ *(Mm) Dse*^–^*^/^*^–^ *(Mm)*
				Characteristic cutaneous features: skin hyperextensibility, easy bruising, skin fragility with atrophic scars Increased palmar wrinkling	Colonic diverticulae Pneumo(hemo)thorax Nephrolithiasis/Cystolithiasis Hydronephrosis Cryptorchidism in males Strabismus Refractive errors Glaucoma	
Spondylodysplastic (spEDS)	*B4GALT7 B3GALT6*	Galactosyltransferase-I (β4GalT7) Galactosyltransferase-II (β3GalT6)	AR	Short stature (progressive in childhood) Muscle hypotonia (ranging from severe congenital to mild later-onset) Bowing of limbs	Skin hyperextensibility, soft and doughy, thin and translucent skin Pes planus Delayed motor development Osteopenia Delayed cognitive impairment *spEDS-B4GALT7:* Radioulnar synostosis Bilateral elbow contractures Single transverse palmar crease Characteristic craniofacial features Characteristic X-ray findings of skeletal dysplasia Clouded cornea *spEDS-B3GALT6:* Kyphoscoliosis (congenital or early-onset) Joint hypermobility (generalized or restricted to distal joints) Joint contractures (congenital or progressive) Peculiar fingers Characteristic craniofacial features Tooth discoloration, dysplastic teeth Characteristic X-ray findings of skeletal dysplasia Osteoporosis with spontaneous fractures Aortic aneurysm Lung hypoplasia, restrictive lung disease	*b4galt7-KD (Dr) b4galt7^*sgRNA*^ (Dr) b4galt7*^–^*^/^*^–^ *(Dr) b3galt6*^–^*^/^*^–^ *(Dr)*
**e. Disorders of intracellular processes**						
Spondylodysplastic (spEDS)	*SLC39A13*	Zrt/Irt-Like Protein 13 (ZIP13)	AR	Short stature (progressive in childhood) Muscle hypotonia (ranging from severe congenital to mild later-onset) Bowing of limbs	Skin hyperextensibility, soft and doughy, thin and translucent skin Pes planus Delayed motor development Osteopenia Delayed cognitive impairment *spEDS-SLC39A13:* Protuberant eyes with bluish sclerae Hands with finely wrinkled palms Skeletal dysplasia Atrophy of thenar muscles and tapering fingers Hypermobility of distal joints Characteristic X-ray findings of skeletal dysplasia	*Slc39a13-KO (Mm)*
Brittle cornea syndrome (BCS)	*ZNF469 PRDM5*	Zinc Finger Protein 469 (ZNF469) PR Domain Zinc Finger Protein C5 (PRDM5)	AR	Thin cornea with/without rupture Early-onset progressive keratoconus and/or keratoglobus Blue sclerae	Enucleation or corneal scarring as a result of previous rupture Progressive loss of corneal stromal depth High myopia Retinal detachment Deafness (often mixed conductive and sensorineural) Hypercompliant tympanic membranes Developmental dysplasia of hip Hypotonia in infancy (usually mild) Scoliosis Arachnodactyly Hypermobility of distal joints Pes planus, hallux valgus Mild finger contractures Soft, velvety and/or translucent skin	*/ prdm5-KD (Dr) prdm5^*hi61Tg*^ (Dr)*
**f. Disorders of complement pathway**						
Periodontal (pEDS)	*C1S C1R*	Complement C1s (C1s) Complement C1r (C1r)	AD	Severe and intractable early-onset periodontitis Lack of attached gingiva Pretibial plaques Family history of first degree relative who meets clinical criteria	Easy bruising Joint hypermobility, mostly distal Skin hyperextensibility and fragility, wide or atrophic scarring Increased infection rate Hernias Marfanoid facial features Acrogeria Prominent vasculature	*/ /*
**g. Additional EDS variant**						
Classical-like type 2 (clEDS2) (provisional)	*AEBP1*	Adipocyte enhancer-binding protein-1 (AEBP1)	AR	Skin hyperextensibility with atrophic scarring Generalized joint hypermobility Foot deformities Early-onset osteopenia		*Aebp1*^–^*^/^*^–^ *(Mm)*
**Molecularly unsolved EDS forms**						
Hypermobile (hEDS)	*unknown*	unknown	?	Generalized joint hypermobility Systemic manifestations of generalized connective tissue disorder Positive family history Musculoskeletal complaints Exclusion of other EDS types and other GJH-associated conditions [for detailed description of clinical criteria, see Malfait et al. (2017)]		*/*

*AD, autosomal dominant; AR, autosomal recessive. Known EDS-associated genes affect either **(a)** the primary structure and processing of fibrillar collagens (COL1A1, COL1A2, COL3A1, COL5A1, COL5A2, and ADAMTS2); **(b)** collagen folding and cross-linking (PLOD1 and FKBP14); **(c)** myomatrix function and organization (TNXB and COL12A1); **(d)** glycosaminoglycan biosynthesis (B3GALT6, B4GALT7, CHST14, and DSE); **(e)** intracellular processes (SLC39A13, ZNF469 and PRDM5), **(f)** the complement pathway (C1S and C1R), and **(g)** additional EDS variant (AEBP1). Mm: Mus musculus (mouse), Dr: Danio rerio (zebrafish), /: no model available.*

## The Ehlers–Danlos Syndromes, Collagen-Related Disorders

Fibrillar collagens are trimeric proteins consisting of either three identical (homotrimer) or genetically distinct (heterotrimer) polypeptide chains, referred to as pro-α-chains, which contain repeating Gly-Xaa-Yaa triplets, comprising glycine and two other amino acids, and which form a long uninterrupted triple helical domain, flanked by globular carboxy- (C)- and amino- (N-) terminal propeptides.

**FIGURE 1:**
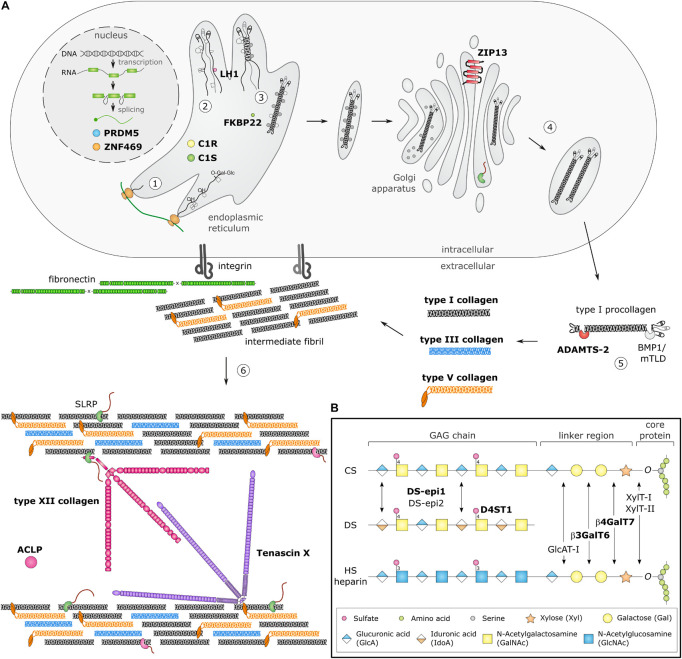
Schematic overview of collagen and glycosaminoglycan (GAG) biosynthesis and collagen fibrillogenesis. Molecules defective in Ehlers-Danlos syndromes (EDS) are highlighted in bold. **(A)** Fibrillar collagen biosynthesis starts with transcription and translation of pro-α-chains (step 1). Nascent pro-α-chains are heavily post-translationally modified by several proline and lysine hydroxylases and galactosyltransferases (step 2). The association of the C-terminal propeptides of three pro-α-chains, initiates triple helix formation which propagates to the N-terminus in a zipperlike fashion and is assisted by several molecular chaperones (step 3). The trimeric procollagen molecules aggregate laterally, are transported in secretory vesicles and are eventually directed to the extracellular environment (step 4). Removal of the N- and C-propeptides, by ADAMTS-2 and BMP-1/mTLD, respectively, results in the formation of a collagen molecule (step 5) that can then assemble into highly ordered striated fibrils. The tissue-specific assembly of collagen fibrils requires the concerted action of several assisting proteins, categorized as organizers, nucleators and regulators (step 6). At the plasma membrane, fibronectin and integrins serve as *organizers* of fibril assembly. Some collagens, such as type V collagen, function as *nucleators*, which initiate immature fibril assembly at the cell surface. Type V collagen co-assembles with type I collagen into heterotypic fibrils with the entire triple helical domain of type V collagen embedded within the fibril, whereas its partially processed N-propeptide domain protrudes to the fibril surface and controls fibrillogenesis by sterically hindering the addition of collagen monomers. The intermediate fibrils are then deposited into the extracellular matrix (ECM). Stabilization of these fibrils is provided by interactions with regulators such as the small leucine-rich proteoglycan (SLRP) decorin, tenascin-X and type XII collagen, which influence the rate of assembly, size and structure of the collagen fibrils. As fibrillogenesis proceeds, fibril growth occurs through linear and lateral fusion of intermediate collagen fibrils which are subsequently stabilized by the formation of covalent intra- and inter-molecular cross-links. **(B)** GAG biosynthesis starts with the synthesis of a proteoglycan core protein which is subsequently modified by several Golgi-resident enzymes. Initially, a common linker region containing four monosaccharides is formed. Biosynthesis of this tetrasaccharide linker region starts with the stepwise addition of a xylose (Xyl) residue to a specific serine residue of the core protein, catalyzed by xylosyltransferase-I and II (XylT-I/-II). Subsequently, two galactose (Gal) residues are added by galactosyltransferase-I (GalT-I or β4GalT7) and galactosyltransferase-II (GalT-II or β3GalT6). Finally, the addition of a glucuronic acid (GlcA), catalyzed by glucuronosyltransferase-I (GlcAT-I) completes the formation of the linker region. The alternating addition of either N-acetyl-glucosamine (GlcNAc) or N-galactosyl-glucosamine (GalNAc) and GlcA defines the composition of the GAG-chain and subdivides proteoglycans into heparan sulfate (HS) proteoglycans and chondroitin sulfate (CS)/dermatan sulfate (DS) proteoglycans. The GAG-chains are then further modified by epimerization and sulfation. DS synthesis necessitates the epimerization of GlcA towards iduronic acid (IdoA), which is catalyzed by DS epimerases–1 and -2 (DS-epi1 and DS-epi2). Subsequently, dermatan 4-*O*-sulfotransferase-1 (D4ST1) is able to catalyze 4-*O*-sulfation of GalNAc, thereby preventing back-epimerization of the adjacent IdoA.

Biosynthesis and fibrillogenesis have been extensively studied for the prototypic and most abundant type I collagen and are complex processes requiring the orchestrated action of several modifying enzymes, chaperones and ECM molecules (Figure 1A; Myllyharju and Kivirikko, 2004; Canty and Kadler, 2005). In brief, nascent pro-α-chains are heavily post-translationally modified, followed by association of the C-terminal propeptides of three pro-α-chains which assemble into a trimeric procollagen molecule propagating to the N-terminus in a zipperlike fashion. These procollagens are subsequently transported to the extracellular environment and N- and C-propeptides are proteolytically removed, resulting in the formation of a collagen molecule that can then assemble into highly ordered cross-striated fibrils and fibers (Birk and Brückner, 2010). Collagen fibrils are usually composed of different fibrillar collagen types and collagen fibril assembly occurs in a tissue-specific way, requiring the concerted action of several assisting proteins, proposed to be categorized into three general classes: organizers (e.g., fibronectin, integrins), nucleators (e.g., type V collagen) and regulators [e.g., small leucine-rich proteoglycans (SLRPs), fibril-associated collagens with interrupted triple helices (FACIT), and glycoproteins] (Kadler et al., 2008). As fibrillogenesis proceeds, fibril growth occurs through linear and lateral fusion of intermediate collagen fibrils which are subsequently stabilized by the formation of intra- and inter-molecular cross-links (Birk, 2001; Canty and Kadler, 2005; Greenspan, 2005).

Insights into the function of some of the proteins involved in these processes have been irrevocably intertwined with the identification of genetic defects in human disorders, including EDS, but also with the study of animal models.

## Animal Models Mimicking Ehlers–Danlos Syndromes

Animals have been extensively used as experimental models for human diseases in biomedical research and have successfully provided tools for studying the function of a particular protein, understanding pathogenic cellular and molecular mechanisms in relevant tissues as well as the preclinical development and testing of therapeutic options for a wide variety of human diseases (Okechukwu, 2018). Traditionally, mainly rodents [mice (*Mus musculus*) and rats (*Rattus norvegicus*)] were used as experimental animals due to their anatomical, physiological and genetic similarity to humans. More recently, zebrafish (*Danio rerio*) have gained popularity in biomedical research and have emerged as a promising animal model with several advantages, such as small size, easy genetic manipulation, high fecundity, external fertilization, large offspring, transparency of rapidly developing embryos, and low maintenance cost (Kimmel, 1989; Lieschke and Currie, 2007).

**FIGURE 2:**
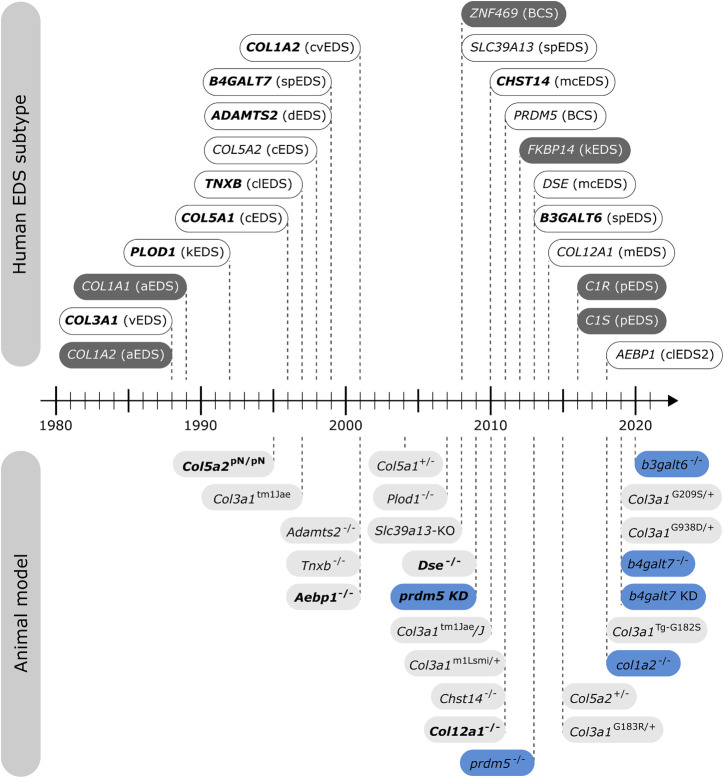
Timeline illustrating the first identification of molecular defects in human EDS (above timeline) and the first description of engineered animal models targeting an EDS-associated gene (below timeline). Human genes associated with EDS without a corresponding animal model are indicated in dark gray. Note that for some human EDS subtypes, biochemical and/or ultrastructural findings preceded the identification of the molecular defect. Mouse models are depicted in light gray and zebrafish models in blue. Bold indicates what was first, either the identification of the human disease gene or the generation of the engineered animal model.

In nature, several spontaneously occurring animals presenting EDS features exist. Additionally, over the past two decades, engineered animal models of EDS have been developed (Figure 2), which have increased our knowledge about the phenotype and our understanding of the mechanisms underlying these conditions.

The aim of this narrative review is to provide an overview of available animal models with defects in EDS-related genes, describe how these animals recapitulate the human phenotype, and how these models have assisted the research community in broadening our knowledge on these conditions and respective protein defects. Therefore, we searched PubMed for a combination of the individual EDS-related genes, combined with the following terms: “Ehlers–Danlos syndrome,” “EDS,” “mouse,” “animal,” and “model.”

### Engineered Animal Models of Ehlers–Danlos Syndromes

**FIGURE 3:**
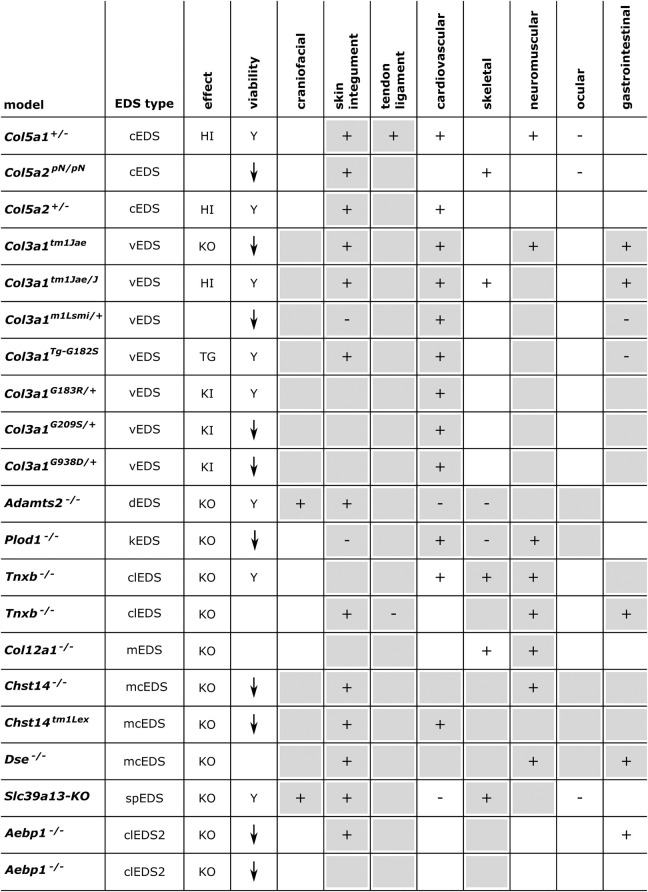
Phenotypic characteristics of mouse models with defects in EDS-associated genes. The presence or absence of a phenotype in mice is indicated with “+” or “-”, respectively, when investigated. A detailed description of the murine phenotypes can be found in Supplementary Table 1. The major and minor clinical characteristics in human EDS patients as defined in the *International EDS Classification*, published in 2017, are indicated with a gray background (Malfait et al., 2017). HI, haploinsufficiency; KO, (homozygous) knockout; TG, transgenic; KI, knock-in; Y, viable; ↓, decreased survival rate.

**FIGURE 4:**
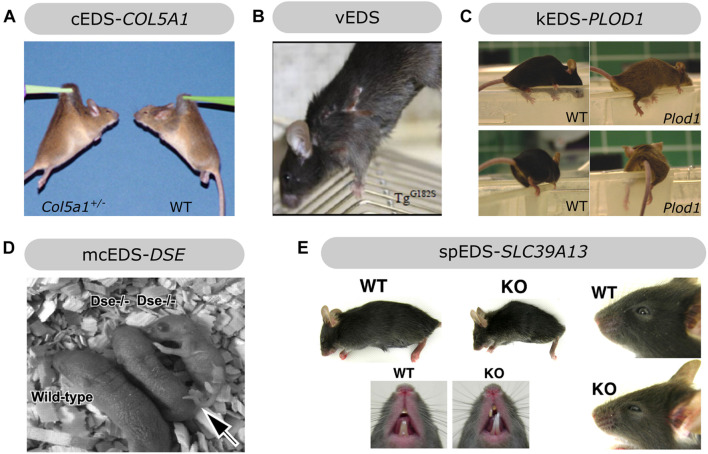
Overview of the phenotypic findings in some mouse models of EDS. **(A)** *Col5a1*^+/–^ mouse model of cEDS. Images adapted from Wenstrup et al. (2006). **(B)** *Col3a1*^*Tg*–*G*182*S*^ mouse model of vEDS. Image adapted from D’hondt et al. (2018). **(C)** *Plod1*^–/–^ mouse model of kEDS-*PLOD1*. Images adapted from Takaluoma et al. (2007). **(D)** *Dse*^–/–^ mouse model of mcEDS-*DSE*. Image adapted from Maccarana et al. (2009). **(E)** *Slc39A13*-KO mouse model of spEDS-*SLC39A13*. Images adapted from Fukada et al. (2008). Images depicted in **(A,C,D,E)** were used under the Creative Commons License and image B with license number 5090710991420.

**FIGURE 5:**
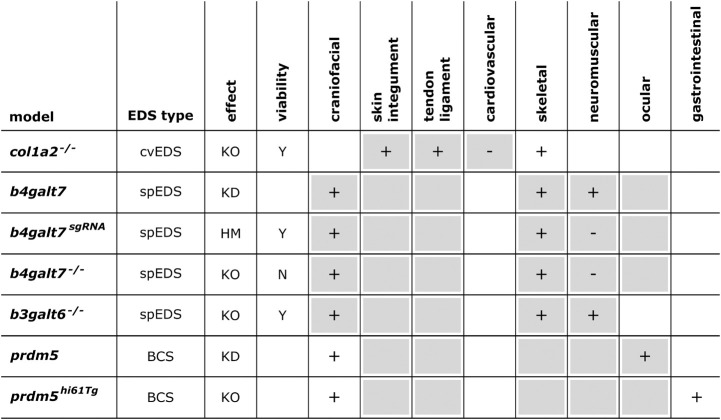
Phenotypic characteristics of zebrafish models with defects in EDS-associated genes. The presence or absence of a phenotype in the zebrafish model is indicated with “+” or “-”, respectively, when investigated. A detailed description of the zebrafish phenotypes can be found in Supplementary Table 2. The major and minor clinical characteristics in humans EDS patients as defined in the *International EDS Classification*, published in 2017, are indicated with a gray background (Malfait et al., 2017). BCS, brittle cornea syndrome; KO, knockout; KD, (morpholino-based) knockdown; HM, hypomorphic; Y, viable; N, not viable.

**FIGURE 6:**
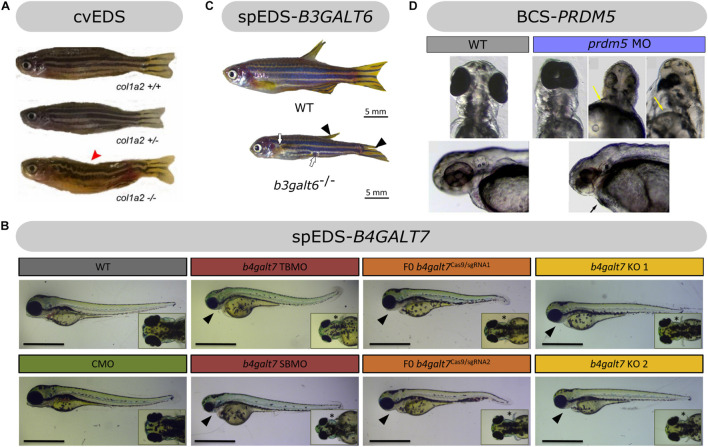
Overview of the phenotypic findings in zebrafish models of EDS. **(A)** Adult *col1a2*^–/–^ zebrafish model of cvEDS. Images adapted from Gistelinck et al. (2018). **(B)** Larval *b4galt7* morphant, crispant and knockout models of spEDS-*B4GALT7*. Scale bars: 1 mm. Images adapted from Delbaere et al. (2019a). **(C)** Adult *b3galt6*^–/–^ zebrafish model of spEDS-*B3GALT6*. Images adapted from Delbaere et al. (2020). **(D)** Morpholino (MO)-based knockdown of *prdm5* in zebrafish larvae. Images adapted from Meani et al. (2009). Images depicted in **(A,C,D)** were used under the Creative Commons License and image B with license number 5078981221357.

Below, we provide an overview of published mouse and zebrafish models with defects in EDS-associated genes, organized according to the underlying genetic and pathogenetic mechanisms as defined in the 2017 EDS classification (Malfait et al., 2017). A detailed summary of the available models is presented in Figures 3, 4 and Supplementary Table 1 for mouse models and Figures 5, 6 and Supplementary Table 2 for zebrafish models. Although other (often conditional) mouse models affecting an EDS-related gene have been reported, an extensive description of these models is beyond the scope of this review (for a brief summary, see Supplementary Information).

#### Models Affecting the Primary Structure and Processing of Fibrillar Collagens

##### Models of Classical Ehlers–Danlos Syndrome—Defects in Type V Collagen

Classical EDS (cEDS) is the most common, molecularly defined EDS subtype. It is characterized by generalized joint hypermobility, skin hyperextensibility and skin fragility, manifesting as easy splitting of the skin upon minor trauma, which, in combination with delayed wound healing, results in the formation of widened atrophic scarring (Bowen et al., 2017). It is inherited in an autosomal dominant fashion and about 90% of cEDS patients harbor a heterozygous mutation in either the *COL5A1* or *COL5A2* gene, encoding the pro-α1- and pro-α2-chain of type V collagen, respectively. The majority of these mutations result in a non-functional *COL5A1* allele and give rise to *COL5A1* haploinsufficiency with reduced type V collagen protein levels, while mutations in *COL5A1* or *COL5A2* that lead to a structural defect (e.g., glycine substitution or in-frame exon skip) have a dominant negative effect (Symoens et al., 2012; Colman et al., 2021). The most common isoform of type V collagen is the [α1(V)]_2_α2(V) heterotrimer found in skin, tendon, ligaments, cornea, and bone, where it forms heterotypic fibrils with type I collagen. Other isoforms, including the embryonic [α1(V)]_3_ homotrimer and the α1(V)α2(V)α3(V) heterotrimer mainly found in placenta, are less abundant. Although type V collagen is quantitatively of low abundance (about 2–5% of total collagen in bone, tendon, and dermis), it plays a key role during collagen fibrillogenesis by initiating fibril assembly and regulating heterotypic type I/V collagen fibril diameter through its partially retained α1(V)-N-propeptide that protrudes beyond the fibril surface (Birk, 2001; Wenstrup et al., 2004).

Several murine models have been generated to study the functional and regulatory roles of type V collagen during collagen fibrillogenesis, and as such these models also provide insights into cEDS pathogenesis.

###### Col5a1 Haploinsufficient Mice

Complete absence of the pro-α1(V)-chains in homozygous *Col5a1* knockout *(Col5a1*^–/–^*)* mice was shown to result in lethality around embryonic day 10 due to cardiovascular failure. Ultrastructural evaluation of *Col5a1*^–/–^ embryos using TEM revealed the virtual absence of predermal mesenchymal fibril formation despite the presence of normal amounts of type I collagen. These findings revealed a critical regulatory role of type V collagen in the initiation of early fibril formation and nucleation of type I collagen fibril assembly during early murine embryogenesis (Wenstrup et al., 2004). Complete loss of the pro-α1(V)-chains has not been reported in humans, most probably because it is lethal.

Heterozygous *Col5a1* knockout (*Col5a1*^+/–^) mice are viable and mimic the most common molecular defect associated with cEDS, i.e., *COL5A1* haploinsufficiency, leading to approximately 50% reduction in type V collagen content (Wenstrup et al., 2006). Similar to cEDS patients, the skin of *Col5a1*^+/–^ mice was hyperextensible with decreased tensile strength (Figure 4A). Ultrastructural analysis showed decreased fibril density in the subscapular dermis, correlating with the decreased collagen content, and consistent with dysfunctional regulation of collagen fibril nucleation when type V collagen is limited. Additionally, two different fibril subpopulations were observed: relatively normal symmetrical fibrils with slightly larger diameters, and very large, structurally aberrant fibrils with irregular contours, so called “cauliflower” fibrils, representing an unregulated assembly of type I collagen which virtually lacks type V collagen and disrupted lateral fibril growth (Wenstrup et al., 2004, 2006). These ultrastructural abnormalities are highly reminiscent of the alterations in dermis of cEDS patients (Vogel et al., 1979). Most *Col5a1*^+/–^ mice older than 6 months developed spontaneous, non-healing wounds indicative of skin fragility (DeNigris et al., 2015). Wound repair studies demonstrated slower *in vivo* closure of a subscapular skin wound in *Col5a1*^+/–^ mice. Subsequent *in vitro* studies on dermal fibroblasts from *Col5a1*^+/–^ mice showed that this impaired wound healing is likely attributed to an interplay of decreases in proliferation rate, attachment properties to components of the wound ECM (e.g., types I and III collagen and fibronectin) and migration capacity of *Col5a1*^+/–^ fibroblasts (DeNigris et al., 2015). The latter finding is consistent with *in vitro* scratch assays showing delayed migration of dermal fibroblasts from cEDS patients with *COL5A1* haploinsufficiency (Viglio et al., 2008; DeNigris et al., 2015).

Although no overt joint hypermobility, another major feature of human cEDS, was described in *Col5a1*^+/–^ mice (Wenstrup et al., 2006), biomechanical analysis of the flexor digitorum longus (FDL) and patellar tendons showed reduced tensile strength, suggesting increased elasticity. Ultrastructural analyses of FDL tendon showed unexpectedly mild abnormalities in *Col5a1*^+/–^ mice with less regular cross-sectional profiles and smaller diameter of collagen fibrils (Wenstrup et al., 2011). TEM of patellar tendon showed two distinct subpopulations of smaller and larger diameter fibrils with mostly normal circular fibril cross-section profiles but with larger diameter fibrils in Col5a1^+/−^ mice (Johnston et al., 2017). Functional and structural tendon pathology has also been described in the patellar tendon of cEDS patients with low tendon stiffness, and abnormal ultrastructural findings with various amounts of large and irregular collagen fibrils combined with apparently normal fibrils (Nielsen et al., 2014). Tendons from *Col5a1*^+/–^ mice showed diminished mechanical recovery potential following bilateral patellar tendon injury with smaller fibril sizes 6 weeks post-injury, indicating further failure in the healing response in *Col5a1*^+/–^ tendon. These findings point to a role for type V collagen in healing and recovery following injury (Johnston et al., 2017).

*Col5a1*^+/–^ mice also displayed a vascular phenotype with decreased aortic stiffness and breaking strength, however not associated with ruptures or sudden, premature death (Wenstrup et al., 2006). The heart was morphologically normal, but immunohistochemical analysis revealed increased deposition of type I and III collagen in mitral and aortic valves and ventricular myocardium, supposedly as a compensatory mechanism for disturbed fibrillogenesis due to reduced type V collagen content (Lincoln et al., 2006). Although these findings indicate the importance of type V collagen in vascular and cardiac structure, integrity and/or function, cardiovalvular problems are rarely of clinical significance and severe or life-threatening arterial manifestations are only sporadically observed in cEDS patients (Bowen et al., 2017; Malfait, 2018; Angwin et al., 2020; Colman et al., 2021).

While type V collagen is a quantitatively minor collagen in most tissues, it accounts for 10–20% of total corneal collagen (Birk, 2001). Cornea of *Col5a1*^+/–^ mice were grossly normal without opacity but showed reduced stromal thickness and a reduced collagen content. At the ultrastructural level, stromal collagen fibril density was decreased, with a single population of consistently larger, cylindrically shaped fibril diameters but unaltered fibril packing (Segev et al., 2006). Intriguingly, mild corneal abnormalities, (e.g., thin and steep but transparent corneas) have been described in few cEDS patients (Segev et al., 2006).

Our group recently reported that both male and female *Col5a1*^+/–^ mice are hypersensitive to mechanical stimuli applied to the hind paws and abdominal area but not to thermal stimuli. Additionally, female *Col5a1*^+/–^ mice showed altered climbing behavior with decreased grip strength. Visualization of Na_V_1.8-positive nociceptors in the glabrous skin of the footpad revealed a decreased intraepidermal nerve fiber density, with fewer nerves crossing the dermis-epidermis junction in *Col5a1*^+/–^ mice (Syx et al., 2020).

###### Homozygous Exon 6 Deletion in Murine Col5a2

Prior to the elucidation of the molecular basis of human cEDS, involvement of type V collagen in its pathogenesis was already suggested by mice harboring a homozygous in-frame deletion of exon 6 in the *Col5a2* gene. The resulting so-called pN/pN mice (*Col5a2*^*pN/pN*^) lack the amino-terminal telopeptide of the pro-α2(V)-chain (Andrikopoulos et al., 1995). Although initially suggested to result in a structurally abnormal α2(V)-chain with a dominant-negative effect, subsequent analyses revealed that the *Col5a2* deletion resulted in severely reduced pro-α2(V)-chains levels (despite normal *Col5a2* expression levels). As a consequence, compensatory upregulated *Col5a1* expression was seen with the predominant formation of [α1(V)]_3_ homotrimers in skin (Chanut-Delalande et al., 2004). Most homozygous *Col5a2*^*pN/pN*^ mice died during the first 48h of postnatal life, with a survival rate of only 5% past weaning age, mainly due to respiratory problems triggered by varying degrees of lordosis and kyphosis. Surviving *Col5a2*^*pN/pN*^ mice weighed approximately half of the wild-type littermates at 3 weeks of age, attributed to reduced mobility due to the progressive spinal deformities prohibiting nourishment. They displayed severe skin fragility (with multiple scars and bleeding lacerations) and increased stretchability of the skin, reminiscent of EDS. Histological analyses of the skin of *Col5a2*^*pN/pN*^ mice revealed reduced dermal and increased hypodermal thickness, with unusually localized hair follicles in the latter, while ultrastructural analyses demonstrated that dermal collagen fibrils were more disorganized, less tightly packed and heterogeneous in size with areas devoid of banded fibrils (Andrikopoulos et al., 1995; Chanut-Delalande et al., 2004). Dermal fibroblasts derived from *Col5a2*^*pN/pN*^ mice produced a sparse network of disorganized, thin fibrils *in vitro* and also showed increased apoptosis (Chanut-Delalande et al., 2004). These observations underscore the importance of type V collagen and the [α1(V)]_2_α2(V) heterotrimer for proper cell-matrix interactions during skin development. Additionally, pronounced ultrastructural disorganization of collagen fibrils with an overall increased diameter was also seen in the corneal stroma, which seemed thinner in *Col5a2*^*pN/pN*^ mice (Andrikopoulos et al., 1995; Chanut-Delalande et al., 2004).

###### Col5a2 Haploinsufficient Mice

In 2015, a constitutive *Col5a2* knockout (*Col5a2*^–/–^) mouse model was created with complete absence of pro-α2(V)-chains (Park et al., 2015). In contrast to *Col5a2*^*pN*/pN^ mice, homozygous *Col5a2*^–^*^/^*^–^ mice were embryonic lethal around 12 days post conception (dpc) due to cardiovascular insufficiency. In contrast to the absence of fibrils in *Col5a1*^–/–^ embryos, mesenchymal collagen fibrils with abnormally large diameters and abnormal configurations were observed in *Col5a2*^–/–^ embryos, suggesting that [α1(V)]_3_ homotrimers can at least partially compensate for the loss of [α1(V)]_2_α2(V) heterotrimers in the initiation of early embryonic collagen fibril formation.

Whereas heterozygous *Col5a2*^*pN*/+^ mice did not display an overt phenotype, heterozygous *Col5a2*^+/–^ mice representing *Col5a2* haploinsufficiency did survive and showed increased skin extensibility with decreased tensile strength, although less pronounced compared to *Col5a1*^+/–^ mice. Ultrastructural analyses showed only mild collagen fibril abnormalities in the subscapular dermis of *Col5a2*^+/–^ mice with irregular collagen fibril contours mainly seen on longitudinal sections, but without the pathognomonic “cauliflower”-shaped dermal collagen fibrils found in cross-sections of cEDS patients and *Col5a1*^+/–^ mice. Reduced tensile strength and stiffness pointing to increased fragility and elasticity, respectively, were also noted in the aorta of *Col5a2*^+/–^ mice (Park et al., 2015). Additionally, experimentally increasing blood pressure by angiotensin II administration showed an increased incidence, diameter and severity of abdominal aortic aneurysms with more than half of *Col5a2*^+/–^ mice dying of aortic arch dissection and rupture (Park et al., 2017). To date, no cEDS patients with *COL5A2* haploinsufficiency have been reported.

Taken together, *Col5a1*^+/–^ mice mimic human cEDS the best since they represent the major molecular defect, i.e., *COL5A1* haploinsufficiency, and faithfully recapitulate many of the clinical (including pain), biomechanical, morphological and biochemical features seen in cEDS patients (Wenstrup et al., 2006). To date, no animal models harboring structural defects such as glycine substitutions or in-frame exon skips in the triple helical domain of the pro-α1- or pro-α2-chains of type V collagen have been reported.

##### Models of Vascular Ehlers–Danlos Syndrome—Defects in Type III Collagen

Vascular EDS (vEDS) is an autosomal dominant condition mainly characterized by life-threatening complications, including arterial aneurysms, dissections and ruptures, but also bowel perforations/ruptures and ruptures of the gravid uterus, resulting in a reduced life span with a median survival age of 51 years (Pepin et al., 2014; Byers et al., 2017). vEDS patients often have thin, translucent skin, excessive bruising and present with a characteristic facial appearance. To date, the only evidence-based treatment strategy for vEDS that decreases the incidence of arterial rupture is celiprolol, a long-acting β1-receptor antagonist with partial β2-receptor agonist properties used for treatment of hypertension and reducing arterial wall stress, without changing hemodynamic parameters (Ong et al., 2010). Nevertheless, caution is warranted due to some study limitations (Backer and Backer, 2019). The vast majority of vEDS patients harbors a heterozygous defect in the *COL3A1* gene, encoding the pro-α1-chain of type III collagen. Most genetic defects are missense or splice site mutations, introducing a glycine substitution or creating an in-frame exon skip within the triple helical domain, respectively. Mutations leading to *COL3A1* haploinsufficiency were reported in a small proportion (less than 5%) of vEDS patients and are associated with a delayed onset of complications by almost two decades (Pepin et al., 2014). Type III collagen is a homotrimer consisting of three α1(III)-chains, found abundantly in the wall of arteries, gastrointestinal tract, uterus and skin, where it often co-localizes with type I collagen and is thought to regulate fibril diameter (Epstein and Munderloh, 1975).

Several murine models have been generated to study the role of (defective) type III collagen and vEDS pathogenesis.

###### Homozygous Col3a1 Knockout Mice

The first proposed vEDS model was a homozygous *Col3a1* knockout (*Col3a1*^*tm*1*Jae*^) mouse, which had a 5% survival rate at weaning age with most deaths occurring within 48 h after birth of unknown etiology. Surviving homozygous *Col3a1*^*tm*1*Jae*^ mice appeared normal but were 15% smaller than wild-type mice and had a reduced lifespan (6 months), mainly caused by rupture of large blood vessels and occasionally by intestinal rupture. About 60% of surviving homozygous knockout mice displayed spontaneous skin lesions, including large open wounds. TEM analyses of aorta, skin, intestine, liver, heart, and lung showed a reduced number of collagen fibrils in mutant mice with variable fibril diameters of almost twice the size of those in wild-type mice, hinting at a critical role for type III collagen during collagen fibrillogenesis (Liu et al., 1997). The high mortality of this model precludes preclinical studies. Additionally, complete loss of type III collagen in human patients is rare and not associated with a typical vEDS phenotype. Biallelic *COL3A1* mutations result in connective tissue abnormalities with some phenotypic resemblance to vEDS combined with structural brain anomalies. (Plancke et al., 2009; Jørgensen et al., 2014; Horn et al., 2017; Vandervore et al., 2017). In accordance with this human phenotype, the brain of homozygous *Col3a1*^*tm*1*Jae*^ mice showed cobblestone-like cortical malformation with pial basement membrane defects and neuronal overmigration, thereby suggesting a role for type III collagen during brain development (Jeong et al., 2012).

###### Col3a1 Haploinsufficient Mice

Heterozygous *Col3a1* knockout (*Col3a1*^*tm*1*Jae*^/*J*) mice, which model *Col3a1* haploinsufficiency, were initially reported to be phenotypically normal without spontaneous life-threatening vascular or gastrointestinal complications or premature death up to 2 years of age (Liu et al., 1997). However, more detailed analysis revealed some late-onset histological lesions in the aorta with fragmentation of the internal elastic lamina, which aggravated with age (from 9 to 21 months) and were more pronounced in male (88%) versus female (47%) *Col3a1*^*tm*1*Jae*^/*J* mice (Cooper et al., 2010). Additionally, the abdominal aorta had decreased wall strength and stiffness likely due to reduced thickness and collagen content with increased matrix metalloproteinase (MMP) activity, particularly MMP-9 (Cooper et al., 2010; Tae et al., 2012; Goudot et al., 2018). In line with the vascular integrity deficits, experimentally increasing blood pressure by angiotensin II administration increased the susceptibility for thoracic aortic dissection in *Col3a1*^*tm*1*Jae*^/*J* mice (Faugeroux et al., 2013). Long-term treatment of *Col3a1*^*tm*1*Jae*^/*J* mice with doxycycline, a tetracycline antibiotic and nonspecific MMP inhibitor, aimed at strengthening the arterial walls by decreasing ECM degradation by MMPs, attenuated the decrease in aortic collagen content and prevented the development of spontaneous and stress-induced aortic lesions (Briest and Talan, 2011; Briest et al., 2011; Tae et al., 2012). Colons of *Col3a1*^*tm*1*Jae*^/*J* mice showed reduced strength and increased compliance, despite normal histology (Cooper et al., 2010). Bladders of *Col3a1*^*tm*1*Jae*^/*J* mice also showed increased compliance, less densely packed collagen fibrils with a broader diameter distribution on ultrastructure of the lamina propria and decreased neurotransmitter function (Stevenson et al., 2006). *Col3a1*^*tm*1*Jae*^/*J* mice did not present with a clear skin phenotype, but faster experimental wound closure with increased wound contracture was seen in a collagen gel contraction assay with embryonic dermal fibroblasts *in vitro* and *in vivo* in adult mice (>1 year), accompanied by increased myofibroblast differentiation and increased scar tissue formation (Volk et al., 2011). Analysis of the skeleton revealed reduced trabecular bone quantity but no craniofacial abnormalities in *Col3a1*^*tm*1*Jae*^/*J* mice (Volk et al., 2014) and a bilateral tibial fracture model showed impaired bone formation and altered remodeling during fracture healing in *Col3a1*^*tm*1*Jae*^/*J* mice, without alterations in mechanical bone function (Miedel et al., 2015). These findings suggest a role for type III collagen in both cutaneous and skeletal development and repair. Taken together, haploinsufficient *Col3a1*^*tm*1*Jae*^/*J* mice model only a small subset of human vEDS patients (<5%) with a milder phenotype and although this model shows subclinical, age-dependent evidence for vascular and gastrointestinal fragility, preclinical studies are largely limited by the lack of overt disease.

###### Mice Harboring a Heterozygous In-frame Deletion in Col3a1

Another mouse model for vEDS was identified serendipitously during a gene-targeting study. Although this model was initially reported to harbor a 185 kb deletion affecting the promotor region and exons 1–39 of *Col3a1* thereby introducing a premature termination codon (Smith et al., 2011), subsequent molecular characterization revealed an in-frame deletion of exons 33–39 (Dubacher et al., 2019). Whereas homozygous *Col3a1*^*m*1*Lsmi*/*m*1*Lsmi*^ mice were embryonic lethal, heterozygous *Col3a1*^*m*1*Lsmi*/+^ mice displayed sudden death due to acute aortic dissection, mostly between 4 and 10 weeks of age, with incomplete penetrance (28%) and two times more frequent in males versus females. Prior to aortic dissection, heterozygous *Col3a1*^*m*1*Lsmi*/+^ mice were indistinguishable from wild-type mice, without hypertension, aneurysms or dilatation of the ascending aorta *in vivo* and little evidence of cardiovascular dysfunction. However, collagen content in the media of the thoracic aorta was reduced in *Col3a1*^*m*1*Lsmi*/+^ mice and ultrastructural examination of the thoracic aorta revealed inconsistencies in the elastic laminae structure, tearing of the smooth muscle layer, and a reduced number of collagen fibrils with more variable and larger diameters in the adventitia, similar to findings in the skin (Smith et al., 2011; Dubacher et al., 2019). The thoracic aorta of *Col3a1*^*m*1*Lsmi*/+^ mice had a lower maximal tensile strength compared to wild-type mice. This biomechanical integrity test of the thoracic aorta was used as a readout for therapeutic intervention and was ameliorated following treatment with the β-blocker celiprolol and MMP inhibitor doxycycline, but not with the angiotensin II receptor type 1 antagonist losartan or β-blocker bisoprolol (Dubacher et al., 2019; Gorosabel et al., 2019). Overall, this model appears to mimic only the vascular features of vEDS, which are more severe compared to *Col3a1*^*tm*1*Jae*^/*J* haploinsufficient mice, without overt evidence of skin fragility, gastrointestinal or uterus rupture (Smith et al., 2011).

###### Transgenic Col3a1 Mice

Since glycine substitutions in the pro-α1(III) triple helical domain are the main molecular defects found in the vast majority of vEDS patients (Pepin et al., 2014), our group developed a transgenic *Col3a1* (*Col3a1*^*Tg*–*G*182*S*^) mouse model overexpressing a typical glycine substitution, p.(Gly182Ser) (D’hondt et al., 2018), corresponding to the most frequently reported human missense mutation [p.(Gly183Ser)] in type III collagen (Dalgleish, 1998). The skin of *Col3a1*^*Tg*–*G*182*S*^ mice was thin and easily torn when handled. At 13–14 weeks of age all male (but not female) *Col3a1*^*Tg*–*G*182*S*^ mice developed spontaneous transdermal wounds, requiring euthanasia before any major vascular manifestations occurred (Figure 4B; D’hondt et al., 2018). These wounds were reminiscent of the dermal phenotype seen in surviving homozygous *Col3a1*^–/–^ mice (Liu et al., 1997). Total collagen content and tensile strength of 12-week-old *Col3a1*^*Tg*–*G*182*S*^ mice were severely reduced in the abdominal skin and the thoraco-abdominal aorta with reduced thickness of the adventitia of the aorta, indicative of cutaneous and vascular fragility as seen in vEDS patients. Ultrastructural analysis revealed a reduced amount of collagen fibrils in the dermis and highly variable fibril diameters with a tendency to thicker fibrils in the dermis and the adventitia of the thoracic aorta of *Col3a1*^*Tg*–*G*182*S*^ mice. The latter also showed abnormal distribution and morphology of smooth muscle cells and reduced contact with the elastic lamina. These clinical and ultrastructural abnormalities were not detected in a control line overexpressing wild-type type III collagen (*Col3a1*^*Tg–WT*^). Overall, these findings confirmed a key role for type III collagen during collagen fibrillogenesis in the dermis and vasculature (D’hondt et al., 2018).

###### Heterozygous Col3a1 Knock-in Mice

Two groups independently created heterozygous *Col3a1* knock-in mouse models harboring different glycine substitutions. The *Col3a1*^*G*183*R*/+^ mouse model shows spontaneous thoracic aortic rupture resulting in a 50% mortality rate at 24 weeks of age which was lower in female (25%) compared to male (60%) mice (Fontaine et al., 2015). The observed arterial fragility was not preceded by dilatation of the aorta, but reduced abdominal aortic stiffening was observed (Fontaine et al., 2015; Goudot et al., 2018). TEM analysis of the aorta of *Col3a1*^*G*183*R*/+^ mice showed a lower density of collagen fibrils with heterogeneous diameters and dilated endoplasmic reticulum in adventitial fibroblasts (Fontaine et al., 2015). Recently, two additional murine *Col3a1* knock-in models were created, *Col3a1*^*G*209*S*/+^ and *Col3a1*^*G*938*D*/+^, harboring helical glycine substitutions previously identified in vEDS patients (Bowen et al., 2019). Both models suffer from sudden premature death due to spontaneous aortic rupture occasionally accompanied by dissection of the proximal descending thoracic aorta but without aneurysms. As expected from the location of the substituted glycine and the assembly of type III procollagen from the C- to N-terminus (Mizuno et al., 2013), *Col3a1*^*G*938*D*/+^ mice showed a more severe phenotype, including smaller body size, smaller aortas with decreased collagen content and a median survival of 45 days compared to 400 days for *Col3a1*^*G*209*S*/+^ mice. Histology of the aortic wall revealed occasional elastic fiber breaks and TEM of the proximal descending thoracic aorta showed disrupted elastic lamellar units with thickened elastic fibers with a moth-eaten appearance, disarray of vascular smooth muscle cells (VSMCs) and paucity of collagen fibrils in the space between VSMCs and elastic fibers in both models. Collagen fibrils in the aortic media of *Col3a1*^*G*938*D*/+^ mice had a wide variation in diameter and were generally smaller whereas adventitial fibroblasts showed a dilated endoplasmic reticulum. Pharmacological blood pressure reduction using losartan, propranolol, atenolol and amlodipine had no impact on survival rate of the severe *Col3a1*^*G*938*D*/+^ model. Surprisingly, and in contrast to the ameliorated biomechanical findings in *Col3a1*^*m*1*Lsmi*/+^ aortas (Dubacher et al., 2019) and decreased arterial rupture incidence in vEDS patients (Ong et al., 2010), celiprolol accelerated death from aortic dissection in both *Col3a1*^*G*209*S*/+^ and *Col3a1*^*G*938*D*/+^ mice. Transcriptome profiling of the descending thoracic aorta revealed that the vascular rupture risk is mediated by excessive PLC/IP_3_/PKC/ERK signaling and pharmacological attenuation of this pathway by inhibition of IP_3_ (hydralazine), PKCβ (ruboxistaurin), or MEK/ERK (cobimetinib) increased survival in the severe *Col3a1*^*G*938*D*/+^ model. Furthermore, the increased risk for life-threatening vascular events associated with pregnancy and puberty, also seen in vEDS patients (Murray et al., 2014; Byers et al., 2017), was shown to be rescued by inhibiting oxytocin signaling (by removal of pups or oxytocin receptor antagonist), MEK (trametinib) or IP_3_ (hydralazine) in mild *Col3a1*^*G*209*S*/+^ female mice and androgen signaling (with androgen receptor antagonists bicalutamide or spironolactone) in *Col3a1*^*G*938*D*/+^ mice, respectively.

In summary, vEDS is currently the only EDS subtype for which mice are available with *Col3a1* haploinsufficiency, a genomic multi-exon deletion or a glycine substitution (knock-in). Strikingly, sexual dimorphism is seen in almost all of the described vEDS models, with greater phenotypic severity in male mice compared to female mice, reflecting what is seen in vEDS patients (Pepin et al., 2014). Furthermore, vEDS mouse models are the only preclinical models in which pharmacological studies have been performed (summarized in Supplementary Table 3).

##### Models of Rare Ehlers–Danlos Syndrome Subtypes—Defects in Type I Collagen

Although the majority of heterozygous mutations in *COL1A1* or *COL1A2*, encoding the pro-α1- and pro-α2-chain of type I collagen, respectively, result in the brittle bone disorder osteogenesis imperfecta (OI), specific type I collagen defects can also give rise to rare EDS subtypes.

Biallelic *COL1A2* mutations resulting in complete loss of pro-α2(I)-chains are associated with the cardiac-valvular EDS subtype (cvEDS). The clinical hallmarks of cvEDS include severe and progressive cardiac-valvular problems, combined with variable skin hyperextensibility, atrophic scarring, and joint hypermobility (Brady et al., 2017). A recent study from our team focusing on type I collagen defects in zebrafish, described a *col1a2* knockout (*col1a2*^–/–^) zebrafish lacking pro-α2(I)-chains as the first zebrafish mutant for EDS (Figure 6A; Gistelinck et al., 2018). The skin of *col1a2*^–/–^ zebrafish showed a disturbance of the typical stripe pattern, increased fragility and 50% reduction in dermal thickness. Consistent with these findings, biomechanical testing revealed decreased strength of the soft connective tissues, which is in line with the human phenotype. *col1a2*^–/–^ zebrafish also displayed marked kyphosis, most likely as a consequence of local distortion and dislocation of the intervertebral ligament in parts of the vertebral column. Additionally, a mild reduction of bone thickness and mineralization in the vertebral column was observed in *col1a2*^–/–^ zebrafish. These findings are consistent with the presence of joint dislocations and the lack of a severe skeletal phenotype in cvEDS patients (Malfait et al., 2006). Despite the prominent propensity for cardiac-valvular disease in human patients, initial histological analysis of the adult heart from *col1a2*^–/–^ mutants did not show overt morphological abnormalities of the cardiac valves and cardiac function and blood flow was normal in *col1a2*^–/–^ larvae. More in-depth studies are necessary to study the effect of absent pro-α2(I)-chains on the zebrafish cardiac morphology and functioning.

##### Models of Dermatosparaxis Ehlers–Danlos Syndrome—Defects in the Procollagen N-Proteinase

Dermatosparaxis was first described in cattle showing severe fragility of the skin and was the first collagen disorder that was characterized at the ultrastructural and biochemical level in the animal world (Lapière et al., 1971; Hanset and Lapiere, 1974). Human dermatosparaxis EDS (dEDS) is an autosomal recessive condition clinically hallmarked by extreme skin fragility with excessive skin folds at the wrists and ankles, severe bruisability and growth retardation. Patients have a typical facial gestalt with prominent and protuberant eyes with puffy, edematous eyelids, excessive periorbital skin, large fontanels and/or wide cranial sutures, a hypoplastic chin and bluish or grayish sclerae. Animal dermatosparaxis and human dEDS are caused by biallelic mutations in *ADAMTS2*, encoding a disintegrin and metalloproteinase with thrombospondin-motifs Type 1 Motif-2 (ADAMTS-2) (Malfait et al., 2017). ADAMTS-2 is the most prominent type I procollagen N-proteinase but can also cleave the N-propeptide of types II, III, and V procollagen chains and possibly also other substrates (Colige et al., 2005).

A murine homozygous knockout model of *Adamts2* (*Adamts2*^–/–^) was generated. At birth, *Adamts2*^–/–^ mice were indistinguishable from their wild-type littermates. Around the age of two months, *Adamts2*^–/–^ mice developed triangular facies with a shorter snout, had less dense hair with thinner hair follicles, but lacked craniofacial abnormalities or growth retardation, unlike dEDS patients (Li et al., 2001; Goff et al., 2006). Additionally, the skin of *Adamts2*^–/–^ mice felt thinner and softer and was extremely fragile resulting in easy tearing upon handling. While TEM analysis of the skin of 2-day-old *Adamts2*^–/–^ mice was unremarkable, 2-month-old *Adamts2*^–/–^ mice showed collagen fibrils with an unusually curled morphology, a finding consistent with the “hieroglyphic” appearance of cross-sectional collagen fibrils in human dEDS patients (Colige et al., 2004). Although the cutaneous features of *Adamts2*^–/–^ mice largely mimic findings in human dEDS skin, age-dependent changes have not been reported in dEDS patients or animal dermatosparaxis. Histological and/or ultrastructural evaluation of cartilage, skeleton and aorta of *Adamts2*^–/–^ mice were unremarkable (Li et al., 2001; Goff et al., 2006). Only mild dental changes were noted in *Adamts2*^–/–^ mice, with normal incisors but subtle loss of the surface contour of the molar teeth, which contrasts the rather severe secondary dentition abnormalities (e.g., micro- and hypodontia) seen in dEDS patients (Goff et al., 2006; Brady et al., 2017). *Adamts2*^–/–^ mice had abnormal lungs, characterized by decreased parenchymal density, with a pseudo-emphysematous appearance, but without inflammatory exudate or obvious fibrosis. Collagen fibrils in the lung appeared unaffected on TEM (Goff et al., 2006). Female *Adamts2*^–^*^/^*^–^ mice were fertile, but male mice were sterile, pointing to an unexpected role for ADAMTS-2 in maturation of spermatogonia (Li et al., 2001). To date, no pregnancies have been reported in affected individuals and no information on reproduction is available (Brady et al., 2017).

Notably, not all type I collagen-rich tissues of *Adamts2*^–/–^ mice (and human dEDS) are affected to the same degree. Although pro-α(I)-chains with a retained N-propeptide were seen in skin extracts of *Adamts2*^–/–^ mice as expected, fully processed mature α-chains were also observed. Similar findings were obtained for type II (pro)collagen in cartilage (Li et al., 2001), and types I and III (pro)collagen in aorta and lung (Goff et al., 2006) of *Adamts2*^–/–^ mice, thereby indicating that other enzymes (e.g., ADAMTS-3 and ADAMTS-14) can, at least partially, compensate for the loss of ADAMTS-2, in a tissue-specific way (Goff et al., 2006).

Of interest, two additional murine *Adamts2* knockout models, *Adamts2*^Δ28^ and *Adamts2*^Δ245^, harboring a 28 and 245 bp genomic deletion, respectively, were recently established to investigate the proteolytic inactivation of the ECM glycoprotein, reelin, by Adamts-2 in the adult brain and did not focus on the associated EDS phenotype, but both models showed fragile skin and dull fur (Yamakage et al., 2019). This is the first report highlighting a neuronal function for ADAMTS-2 in addition to its role in collagen biosynthesis and connective tissue integrity.

#### Models Affecting Collagen Folding and Cross-Linking

##### Models of Kyphoscoliotic Ehlers–Danlos Syndrome—Defects in Lysyl Hydroxylase 1

Individuals with kyphoscoliotic EDS (kEDS) mainly suffer from congenital muscle hypotonia, progressive kyphoscoliosis and generalized joint hypermobility. Patients also present with abnormal scarring and easy bruising and have an increased risk of fatal arterial ruptures and, in some patients, ocular fragility. The majority of kEDS patients harbor biallelic mutations in the *PLOD1* gene, encoding lysyl hydroxylase 1 (LH1), which catalyzes hydroxylation of specific helical lysine residues within the pro-α collagen chains (Brady et al., 2017). As a result of LH1 deficiency, lysyl residues are underhydroxylated and hydroxylysyl residues underglycosylated, leading to impaired cross-link formation and mechanical instability of affected tissues (Rohrbach et al., 2011). kEDS-*PLOD1* was the first EDS type to be characterized at the biochemical level (Krane et al., 1972).

A homozygous *Plod1* knockout (*Plod1*^–/–^) murine model for kEDS-*PLOD1* was generated and the resulting *Plod1*^–/–^ mice were viable and fertile but showed gait abnormalities and tired quickly (Figure 4C). They were passive when being handled and felt soft and floppy and their movements were powerless, consistent with muscle hypotonia, which is also observed in kEDS-*PLOD1* patients (Takaluoma et al., 2007). Although the majority of *Plod1*^–/–^ mice survived, 15% of these mice (17% of males and 9% of females) died before the age of 1 year (mostly at 1–4 months of age) due to aortic rupture, a life-threatening complication that can also occur in kEDS-*PLOD1* patients. Although the thickness of the aortic wall was normal in surviving *Plod1*^–/–^ mice, VSMCs appeared to be less regularly ordered and presented with degenerative ultrastructural changes, including vacuolization and mitochondrial swelling. Ultrastructural analysis showed more variation with overall increased collagen fibril diameters in the aorta and some fibrils had irregular contours. Similar ultrastructural alterations were seen in the skin but skin hyperextensibility or fragility were lacking in *Plod1*^–/–^ mice. In contrast to the human phenotype, no kyphoscoliosis was observed in these mice. In line with defective LH1 function, tissue-specific decreases in total hydroxylysine residues were apparent in *Plod1*^–/–^ mice, ranging from 22% in skin to 86% in lung. These findings suggest partial compensation by the two other isoenzymes, LH2 and LH3 (Takaluoma et al., 2007).

Overall, *Plod1*^–/–^ mice seem to only partially capture the human kEDS-*PLOD1* phenotype with the presence of muscle hypotonia and aortic ruptures but without kyphoscoliosis or a clear skin phenotype. The latter might reflect differences in remaining hydroxylysine content between mice (22%) and in human (5%) (Steinmann et al., 2003; Takaluoma et al., 2007).

#### Models Affecting Myomatrix Function and Organization

##### Models of Classical-Like Ehlers–Danlos Syndrome—Defects in Tenascin-X

Classical-like EDS (clEDS) is a rare autosomal recessive condition, characterized by generalized joint hypermobility and skin hyperextensibility with easy bruising but without atrophic scarring. Patients harbor biallelic mutations in the *TNXB* gene, resulting in complete absence of the corresponding tenascin-X (TNX) protein (Brady et al., 2017; Green et al., 2020). TNX is a glycoprotein with an architectural function in the ECM and has been shown to localize with collagen fibrils in tendon and dermis (Valcourt et al., 2015).

Tenascin-X was the first structural protein beyond fibrillar collagens or their modifying enzymes associated with EDS and to investigate its hitherto unknown functions, two groups independently created homozygous *Tnxb* knockout (*Tnxb*^–^*^/^*^–^) mice (Matsumoto et al., 2001; Mao et al., 2002). Although *Tnxb*^–/–^ mice appeared morphologically normal at birth they showed progressive skin hyperextensibility, similar to clEDS patients, accompanied by a decreased ultrastructural density of fibrils with normal size and shape, resulting in a reduced dermal collagen content. Despite near-normal collagen synthesis *in vitro*, *Tnxb*^–/–^ skin fibroblasts showed defective deposition of type I collagen. Together with the ability of TNX to regulate the expression of several ECM molecules, these findings pointed to a regulatory role in collagen fibril deposition (Mao et al., 2002; Minamitani et al., 2004a). Nevertheless, conflicting results have been reported for *Tnxb*^–/–^ skin with normal dermal collagen content (Egging et al., 2007) and increased collagen fibril diameters with normal fibril density (Minamitani et al., 2004b), which might be influenced by murine age, sample site and genetic background. Of note, opposed to the elastin fragmentation in clEDS patients, *Tnxb*^–/–^ mice showed increased dermal elastin density, which is probably less stable (Egging et al., 2006).

In contrast to cEDS, clEDS patients do not present with atrophic scarring or delayed wound healing. *Tnxb*^–/–^ mice mimicked the macroscopically normal wound closure of the dorsal skin *in vivo*, associated with reduced breaking strength (Egging et al., 2007). Subsequent *in vitro* experiments using *Tnxb*^–/–^ mouse embryonic fibroblasts suggested that the near-normal wound healing may be due to accelerated local matrix contraction and tissue remodeling caused by increased activation of MMPs, upregulation of TGF-β1 expression and upregulated collagen synthesis followed by the promotion of cell proliferation and migration (Hashimoto et al., 2018). Together, these findings pointed to a role for TNX during the later phase of wound healing when remodeling and maturation of the ECM occurs (Egging et al., 2007). Additionally, subcutaneous adipose tissue of *Tnxb*^–/–^ mice was thicker and contains an increased amount of triglycerides and an altered fatty acid composition, suggesting a role during lipogenesis (Matsumoto et al., 2004).

Unlike clEDS patients, *Tnxb*^–/–^ mice did not show overt signs of joint hypermobility or ligamentous laxity (Egging et al., 2006), although tail and Achilles tendon displayed ultrastructural changes in collagen fibril density similar to observations in the skin (Mao et al., 2002). *Tnxb*^–/–^ mice did present with mild muscle weakness, histological evidence of myopathy and increased turnover of the ECM in quadriceps muscle (Voermans et al., 2011) as well as altered myofascial force transmission (Huijing et al., 2010), consistent with the mild to moderate muscle weakness observed in clEDS patients (Brady et al., 2017). Histology and TEM studies of the sciatic nerve revealed mildly reduced diameters of myelinated fibers and reduced collagen fibril density in the endoneurium in older, but not young, *Tnxb*^–/–^ mice, which may correspond to axonal polyneuropathy seen in clEDS patients (Matsumoto et al., 2002; Voermans et al., 2009, 2011; Sakai et al., 2017). Additionally, blood vessel formation (but not morphology) was altered in the peripheral nervous system of *Tnxb*^–/–^ mice, with a decreased density of blood vessels with increased diameters (Sakai et al., 2017). Interestingly, *Tnxb*^–/–^ mice were reported to have mechanical allodynia but not thermal hypersensitivity. A chemical pain stimulus with formalin injection in the hind paw evoked an increased pain response during the first (acute) phase (0–5 min) and early during the second (inflammatory) phase (16–30 min) in *Tnxb*^–/–^ mice. Transcutaneous sine wave stimuli elicited a hypersensitive response of myelinated Aδ and Aβ fibers, but not of unmyelinated C fibers. Additionally, *Tnxb*^–/–^ mice showed molecular alterations in the dorsal horn of the spinal cord suggestive of spinal central sensitization (Okuda-Ashitaka et al., 2020).

Additional studies in *Tnxb*^–/–^ mice revealed the presence of reduced femoral bone mass with enhanced osteoclast differentiation and bone-resorbing ability (Kajitani et al., 2019), very mild genito-urinary complications (rectal prolapse in <1%) in female mice compared to uterine and vaginal prolapse seen in clEDS patients (Egging et al., 2008), attenuated stromal neovascularization following cauterization (Sumioka et al., 2018) and gastric dysfunction associated with abnormal gastric sensory function (Aktar et al., 2019). Additionally, *Tnxb*^–/–^ mice showed increased anxiety-like behavior as well as superior sensorimotor coordination and emotional learning and memory, without differences in locomotor or home cage activity (Kawakami and Matsumoto, 2011; Voermans et al., 2011). Cognitive development was not studied in clEDS patients (Brady et al., 2017).

In summary, *Tnxb*^–/–^ mice recapitulate the dermatological, mild muscular findings, and some neurological (including pain) findings of human clEDS but lack the articular aspects. These mice have been instrumental in demonstrating a regulatory role for TNX in a broad range of tissues.

##### Models of Myopathic Ehlers–Danlos Syndrome—Defects in Type XII Collagen

Myopathic EDS (mEDS) is a rare EDS subtype clinically characterized by congenital muscle hypotonia and weakness with delayed motor development, proximal joint contractures in combination with distal joint hypermobility, scoliosis or kyphosis and abnormal scarring (Malfait et al., 2017; Delbaere et al., 2019b). mEDS is caused by mutations in *COL12A1* encoding the pro-α1-chain of type XII collagen, a homotrimer that is the largest member of the FACIT family. Type XII collagen interacts with several ECM molecules (e.g., decorin and tenascin-X) and regulates the organization and mechanical properties of collagen fibrils (Chiquet et al., 2014). The majority of defects identified in *COL12A1* give rise to heterozygous substitutions or in-frame exon skips, while a single biallelic loss-of-function *COL12A1* mutation was identified that results in a more severe phenotype (Hicks et al., 2014; Zou et al., 2014).

Before disease-causing mutations in *COL12A1* were identified, type XII collagen-deficient (*Col12a1*^–/–^) mice were generated. *Col12a1*^–^*^/^*^–^ mice were smaller and exhibited skeletal abnormalities with shorter, more slender and fragile long bones as well as aberrant vertebrae structures (Izu et al., 2011). In depth analyses revealed that the bone phenotype is associated with defective differentiation, organization, polarization, morphology and interactions of *Col12a1*^–/–^ osteoblasts (Izu et al., 2011), thereby affecting cell–cell communication (Izu et al., 2016) and resulting in decreased bone matrix formation with altered quality. Although these skeletal manifestations were not observed in human mEDS, patients have kyphosis and/or scoliosis, comparable to the kyphoscoliosis seen in *Col12a1*^–/–^ mice (Izu et al., 2011; Zou et al., 2014). Together with the identification of *COL12A1* defects in human patients, the phenotype of the *Col12a1*^–/–^ mouse model was revisited. *Col12a1*^–^*^/^*^–^ mice showed evidence of (mild) muscle weakness, delayed transition in muscle fiber-type composition and altered force transmission in the muscle-tendon-bone unit. Ultrastructural studies showed more diffusely dispersed collagen fibrils throughout the endomysium of *Col12a1*^–^*^/^*^–^ muscle (Zou et al., 2014). Given the combination of distal joint hypermobility and proximal contractures in mEDS, tendon development was evaluated in *Col12a1*^–^*^/^*^–^ mice, which also showed decreased collagen fibril packing. Tenocytes from *Col12a1*^–^*^/^*^–^ mice had an altered shape and function with lower type I collagen secretion (Izu et al., 2020). Together, these data indicated the crucial role of type XII collagen in the development and maturation of tendon.

Although all animal studies were performed on homozygous *Col12a1*^–/–^ mice, only two siblings with autosomal recessive mEDS due to complete loss of type XII collagen were identified to date (Zou et al., 2014). Nevertheless, muscle weakness and skeletal abnormalities were observed in both *Col12a1*^–/–^ mice and mEDS patients, but the muscular phenotype seems to be milder in mice compared to patients. To date, phenotypic characteristics of heterozygous *Col12a1*^+/–^ mice have not been reported.

#### Models Affecting Glycosaminoglycan Biosynthesis

Proteoglycans represent major ECM components and play essential roles as structural macromolecules, modulators of cell adhesion and motility, ECM and collagen fibril assembly and signal transduction during development, tissue repair, and angiogenesis. Proteoglycans are composed of a specific core protein substituted with one or more glycosaminoglycan (GAG)-chains (Prydz and Dalen, 2000; Bishop et al., 2007; Couchman and Pataki, 2012). GAG biosynthesis is initiated by the stepwise addition of a tetrasaccharide linker to the core protein followed by the addition of repeating disaccharide units that define the GAG-chain as heparan sulfate (HS), chondroitin sulfate (CS), and/or dermatan sulfate (DS) (Figure 1B).

The first hint for the involvement of proteoglycans in the pathophysiology of EDS came from a mouse model deficient for decorin, the prototypic member of a specific class of proteoglycans, the SLRPs. The resulting decorin knockout (*Dcn*^–/–^) mice had thin and fragile skin with reduced tensile strength and an abnormal ultrastructural morphology of collagen fibrils in skin and tendon with larger and more irregular fibril outlines, thereby highlighting the important regulatory role of decorin during collagen fibrillogenesis (Danielson et al., 1997). Subsequently, knockout mice for other SLRPs, lumican (Chakravarti et al., 1998), dermatopontin (Takeda et al., 2002), or mimecan (Tasheva et al., 2002) also displayed signs of skin laxity and fragility and similar ultrastructural collagen fibril abnormalities as seen in *Dcn*^–/–^ mice (Supplementary Table 4). Given the phenotypic resemblance of these animal models to human EDS, the genes encoding SLRPs were considered good candidates for molecularly unresolved EDS patients, but defects in SLRP core proteins could never be linked with EDS (Malfait et al., 2005). Nevertheless, the subsequent identification of genetic defects in enzymes involved in the synthesis and modification of GAG-chains unequivocally confirmed a role for aberrant proteoglycan biosynthesis in human EDS pathogenesis, but the causal defects compromise proper GAG-formation rather than affecting the core protein.

##### Models of Spondylodysplastic Ehlers–Danlos Syndrome—Defects in Galactosyltransferase-I and -II

Biallelic mutations in the *B4GALT7* and *B3GALT6* genes, encoding the linker enzymes galactosyltransferase-I (β4GalT7) and -II (β3GalT6), respectively, that catalyze subsequent steps of the GAG tetrasaccharide linker region biosynthesis (Figure 1B), give rise to two subtypes of spondylodysplastic EDS (spEDS), spEDS-*B4GALT7* and spEDS-*B3GALT6*. Both subtypes are severe, pleiotropic conditions that are generally characterized by short stature, muscle hypotonia, skeletal abnormalities, skin hyperextensibility and translucency, delayed motor development, and osteopenia. Additionally, subtype specific clinical features can be observed (Table 1; Malfait et al., 2017).

To date, no mouse models with complete β4GalT7- or β3GalT6-deficiency have been reported. The lack of relevant *in vivo* models to investigate the pathogenic mechanisms in these EDS subtypes prompted our group to generate zebrafish models for these conditions (Delbaere et al., 2019a, 2020).

###### Hypomorphic b4galt7 Zebrafish Models

Since reported *B4GALT7* mutations result in a variable reduction, but not complete loss, of β4GalT7 activity (Salter et al., 2016), hypomorphic zebrafish with partial loss of β4GalT7 function were generated to model spEDS-*B4GALT7*, including two different morpholino-based *b4galt7* knockdown (morphant) as well as two mosaic F0 *b4galt7* knockout (crispant) models (Delbaere et al., 2019a). In both models, defective β4GalT7 activity led to hampered GAG biosynthesis with diminished amounts of sulfated GAGs and strongly reduced HS and CS proteoglycan levels at 4 days post fertilization (dpf). Comparable to the short stature seen in spEDS-*B4GALT7* patients, morphant and crispant embryos were smaller than wild-type embryos, mainly attributed to their smaller head. During early development, craniofacial features (e.g., triangular, flat face, and narrow mouth) of human spEDS-*B4GALT7* are mimicked in morphant and crispant *b4galt7* zebrafish, which show craniofacial abnormalities with underdeveloped, misshapen and partly absent cartilage structures (Figure 6B). Cranial neural crest cells are also less abundant and more disorganized in the developing jaw. Additionally, severely reduced to absent mineralized bone structures were observed, with delayed ossification, reminiscent of osteopenia seen in some spEDS-*B4GALT7* patients. Furthermore, the bowing of the pectoral fins in the morphants and crispants corresponds to the bowing of the limbs observed in spEDS-*B4GALT7* patients. Only in the *b4galt7* morphant created with a translation-blocking morpholino compromised muscle function and structure were suggested by deficient hatching from the chorion, a delayed touch-evoked escape response and disturbed filamentous actin patterning in trunk muscle. Nevertheless, despite its presence in only one of the *b4galt7* models, muscle hypotonia and delayed motor development are prominent clinical features in spEDS-*B4GALT7* patients.

###### Homozygous b4galt7 Knockout Zebrafish

Homozygous *b4galt7* knockout (*b4galt7*^–/–^) zebrafish were created for validation purposes and displayed similar craniofacial features as seen in the morphant and crispant models. However, as expected from complete loss of β4GalT7, *b4galt7*^–/–^ embryos and larvae showed a total lack of HS and CS GAGs leading to a more severe bone phenotype with delay or absence of ossification of several bone structures, complete loss of cartilage staining and disorganized chondrocyte stacking in the head. These animals died before the age of 10dpf, confirming that complete loss of β4GalT7 activity is not compatible with life.

Taken together, larval *b4galt7* zebrafish models largely phenocopy the human spEDS-*B4GALT7* phenotype and highlight a key role for β4GalT7 during early development, affecting cartilage, bone and possibly muscle formation. The generation of stable knock-in models harboring (reported) missense variants could overcome the phenotypic variability of *b4galt7* crispants and lethality of the *b4galt7*^–/–^ knockout zebrafish and would allow to study the adult phenotype in more depth (Delbaere et al., 2019a).

###### Homozygous b3galt6 Knockout Zebrafish

In contrast to *b4galt7*^–/–^ zebrafish, homozygous *b3galt6* knockout (*b3galt6*^–/–^) zebrafish did survive to adulthood (Delbaere et al., 2020). *b3galt6*^–/–^ zebrafish showed progressive skeletal malformations with variable severity, including reduced body length, misshapen cranial bones, smaller teeth, and general skeletal dysplasia with kyphosis and scoliosis (Figure 6C), which are reminiscent of the skeletal dysplasia phenotype observed in spEDS-*B3GALT6* patients. Additionally, extra intramembranous bone and bony elements were found on the vertebra as well as a generalized reduction in bone volume and thickness, and relatively increased tissue mineral density. Ultrastructural analysis showed less organized type I collagen fibrils in the intervertebral ligament and the inner layer of the vertebral bone. Additionally, electron dense spots were observed in the vertebral bone, which might be remnants of extrafibrillar mineral aggregates, and could contribute to the increased mineralization and brittleness of the skeleton. This might be related to bone fragility with spontaneous fractures observed in spEDS-*B3GALT6*. Adult *b3galt6*^–/–^ zebrafish displayed interruptions of the horizontal stripes. Ultrastructural analysis of the skin showed a thicker epidermis covering the scales and loosely packed dermal collagen fibrils with increased interfibrillar spaces. The latter findings mimic ultrastructural features of spEDS-*B3GALT6* patients and could be attributed to alterations in the GAG-chains of SLRPs which regulate collagen fibrillogenesis. Consistent with the muscle hypotonia and delayed motor development observed in patients, functional and structural muscle abnormalities were detected in *b3galt6*^–/–^ zebrafish, with lower endurance during a swim-tunnel experiment, thicker endomysium surrounding the muscle fibers and increased sarcomere length on ultrastructure. These phenotypic findings are in accordance with the strongly reduced amounts of CS, DS, and HS disaccharides observed in bone, skin, and muscle of *b3galt6*^–/–^ zebrafish. Surprisingly, low amounts of GAGs were still produced in absence of compensatory upregulation of other galactosyltransferases or linker enzymes, and residual proteoglycans contained an immature linker region consisting of only three instead of four sugars (lacking a galactose residue), thereby presenting a rescue mechanism for a deficiency of one of the linker enzymes. The presence of this non-canonical trilinker region could be confirmed in urine of human spEDS-*B3GALT6* samples (unpublished). As such, *b3galt6*^–/–^ zebrafish largely phenocopy the human spEDS-*B3GALT6* phenotype, including craniofacial dysmorphism, generalized skeletal dysplasia, skin involvement and indications for muscle hypotonia.

Collectively, these studies provided proof-of-concept that zebrafish are good models to study the phenotypic and biomolecular consequences of impaired GAG linker region synthesis and further underscore their usefulness as animal models for EDS research.

##### Models of Musculocontractural Ehlers–Danlos Syndrome—Defects in Dermatan 4-*O*-Sulfotransferase-1 and Dermatan Sulfate Epimerase-1

Musculocontractural EDS (mcEDS) is clinically characterized by multiple congenital malformations including characteristic craniofacial features and contractures as well as progressive multisystemic complications such as skin hyperextensibility and fragility, recurrent dislocations, progressive talipes or spinal deformities, and large subcutaneous hematomas (Malfait et al., 2017). mcEDS is caused by biallelic mutations in either *CHST14* or *DSE*, encoding dermatan 4-*O*-sulfotransferase-1 (D4ST1) and dermatan sulfate epimerase-1 (DS-epi1), respectively. Both enzymes are specifically involved in DS GAG biosynthesis (Figure 1B). In contrast to defects in *B3GALT6* and *B4GALT7* that compromise linker formation and affect the synthesis of both CS/DS and HS proteoglycans, D4ST1- or DS-epi1-deficiency only interferes with formation of DS proteoglycans.

###### Homozygous Chst14 Knockout Mice

The first murine homozygous *Chst14* knockout (*Chst14*^–/–^) model was created to study the roles of DS GAGs during neural development and nerve regeneration (Bian et al., 2011). *Chst14*^–/–^ mice showed reduced viability (8% of offspring). Surviving *Chst14*^–/–^ mice had a normal life span but were smaller and had reduced fertility, kinked tail, tooth malformations and increased skin fragility (Akyüz et al., 2013). *Chst14*^–/–^ mice provided evidence for an overall negative impact of DS on peripheral nerve regeneration (Akyüz et al., 2013) but beneficial activity in central nervous system regeneration (Rost et al., 2016), which could be attributed to differences in levels of DS proteoglycans and their interaction partners between the peripheral nervous system and the central nervous system (Rost et al., 2016). Furthermore, *Chst14*^–/–^ mice showed impaired spatial learning and memory and demonstrated that DS is indispensable for synaptic plasticity in the hippocampus, which might be attributed to impaired Akt/mTOR-signaling (Li et al., 2019).

Subsequently, another homozygous *Chst14* knockout (*Chst14*^–^*^/^*^–^) mouse model was studied that was initially created as part of a largescale effort to create a knockout library for secreted and transmembrane proteins (*Chst14*^*tm*1*Lex*^) (Tang et al., 2010). This *Chst14*^–/–^ model also suffered from perinatal lethality, with a markedly decreased survival of 14.8% at embryonic day 18.5 to 1.3% at adulthood. Placentas from *Chst14*^–^*^/^*^–^ fetuses showed a reduced weight, with smaller vascular diameters in the placental villi (the part of the fetal placenta), and hypoxia- and/or necrotic-like changes in a small subset (6%). TEM analysis demonstrated decreased thickness and fragmentation of basement membranes in the capillaries of the villus (Yoshizawa et al., 2018). These placental vascular abnormalities possibly relate to the high perinatal lethality in *Chst14*^–/–^ mice as well as the development of large subcutaneous hematomas in mcEDS-*CHST14* patients. Changing the genetic background (from mixed C57BL/6-129/SvJ to BALB/c), improved the birth rate for *Chst14*^–/–^ mice to 6.12–18.64% (Shimada et al., 2020). Subsequent analysis of the skin, which is affected in mcEDS-*CHST14* patients, show reduced tensile strength *Chst14*^–^*^/^*^–^ mice and TEM analysis revealed increased intrafibrillar spaces with a decreased density and disorganization of collagen fibrils. Additionally, rod-shaped, linear GAG-chains protruded from collagen fibrils in *Chst14*^–^*^/^*^–^ mice compared to the curved GAG-chains wrapped around the collagen fibrils in wild-type (Hirose et al., 2020), similar to TEM findings for mcEDS-*CHST14* patients (Hirose et al., 2019b). Glycobiological analyses of the skin revealed very low levels of DS disaccharides and an excess of CS disaccharides (Hirose et al., 2020).

The characteristic alterations in GAG-structure and disorganization of the collagen network in de skin are similar to ultrastructural observations in mcEDS-*CHST14* patients, thereby making *Chst14*^–/–^ mice a reasonable model for this EDS subtype although the reduced viability compromises further studies.

###### Homozygous Dse Knockout Mice

Before *DSE* defects were identified in mcEDS patients, homozygous *Dse* knockout (*Dse*^–/–^) mice were already generated. *Dse*^–/–^ mice were smaller, presented with a kinked tail that resolved before 4 weeks of age and had reduced fertility (Figure 4D). The skin of *Dse*^–/–^ mice had reduced tensile strength and sparser loose hypodermal connective tissue on light microscopy despite normal collagen content. TEM analysis revealed dermal collagen fibrils with larger diameters but normal fibril density while *Dse*^–/–^ tail tendons showed only a minor shift towards thicker fibrils with mostly fibrils with normal diameter and no differences in diameter in Achilles tendon (Maccarana et al., 2009). This contrasts the normal ultrastructure of dermal collagen fibrils in mcEDS-*DSE* patients, although morphometric analysis was not performed (Syx et al., 2015). Glycobiological examination revealed a decreased, but not totally absent, DS content in the skin, similar to findings in a mcEDS-*DSE* patient (Müller et al., 2013). The residual DS moieties may be attributed to partial compensation by DS-epi2 activity. Changing the genetic background (from mixed 129SvJ-C57BL/6 to NFR), resulted in *Dse*^–/–^ mice with normal body weights, a kinked tail and some developmental malformations, including an abdominal wall defect with herniated intestines in 16% of *Dse*^–/–^ embryos and neural tube defects (e.g., exencephaly and spina bifida) in 5%. Additionally, *Dse*^–/–^ embryos and newborns had thicker epidermal layers with increased amounts of epidermal basal and spinous layer markers (Gustafsson et al., 2014).

The relatively milder phenotype in *Dse*^–/–^ mice is consistent with the findings in mcEDS-*DSE* patients, which also present an apparently milder phenotype compared to mcEDS due to *CHST14* defects, but only a limited number of patients have been reported so far (Lautrup et al., 2020).

#### Models Affecting Intracellular Processes

##### Models of Spondylodysplastic Ehlers–Danlos Syndrome—Defects in ZIP13

Biallelic mutations in the *SLC39A13* gene, encoding the metal transporter Zrt/irt-like protein 13 (ZIP13), result in a phenotype which clinically overlaps with spEDS due to *B4GALT7* and *B3GALT6* mutations and is therefore grouped within the same clinical entity (Malfait et al., 2017). spEDS-*SLC39A13* patients present with moderate short stature, combined with specific facial characteristics, hypo- or oligodontia, distal joint hypermobility, thin and fragile skin, wrinkled palms, and characteristic radiographic abnormalities (Brady et al., 2017; Malfait et al., 2017; Kumps et al., 2020). spEDS-*SLC39A13* patients display collagen underhydroxylation, despite normal activity of lysyl and prolyl hydroxylases (Fukada et al., 2008; Giunta et al., 2008). ZIP13 is a homodimeric transmembrane protein and putative zinc (Zn^2+^) transporter located on the endoplasmic reticulum/Golgi apparatus which regulates the influx of Zn^2+^ into the cytosol (Bin et al., 2011). Interestingly, studies in *Drosophila melanogaster* revealed that the fruit fly homolog of human ZIP13 exports iron (Fe^2+^) and its depletion might attenuate the action of iron-dependent enzymes such as lysyl hydroxylases, possibly explaining the observed underhydroxylation (Xiao et al., 2014).

Homozygous *Slc39a13* knockout (*Slc39a13-KO*) mice showed growth retardation, progressive kyphosis after 3–4 weeks of age, osteopenia, and abnormal cartilage development (Fukada et al., 2008). Additionally, altered craniofacial features, including sunken and downslanting eyes were apparent as well as dental abnormalities, such as incisor deformities and aberrant dentin formation (Figure 4E). The skin of *Slc39a13-KO* mice was more fragile and showed a thinner dermis with smaller and less densely packed collagen fibrils on ultrastructure, the latter possibly attributed to a decreased dermal DS content (Fukada et al., 2008; Hirose et al., 2015). Subcutaneous adipose tissue was thinner (Fukada et al., 2008; Fukunaka et al., 2017). Corneal stroma showed reduced thickness in *Slc39a13-KO* mice with smaller and more widely spaced collagen fibrils which were more randomly oriented (Fukada et al., 2008; Hirose et al., 2018). Except for severe varicosity in the lower extremities, spEDS-*SLC39A13* patients do not present with cardiovascular symptoms. Similarly, *Slc39a13-KO* mice did not show aneurysms or arterial ruptures, however, abnormalities were observed in the tunica media of the thoracic aorta which could potentially result in increased fragility (Hirose et al., 2019a). *Slc39a13-KO* mice showed evidence for reduced osteoblast activity, impaired chondrocyte and adipocyte differentiation as well as irregular cellular morphology of odontoblasts in the molar tooth, dermal fibroblasts, corneal keratocytes, and aortic VSMCs (Fukada et al., 2008; Fukunaka et al., 2017; Hirose et al., 2018, 2019a). Furthermore, murine studies illustrated dysregulation of the BMP- and TGFβ-signaling pathways in several of these connective tissue forming cells, which might contribute to the observed multisystemic phenotype (Fukada et al., 2008).

Taken together, *Slc39a13-KO* mice display craniofacial, dental, skeletal, dermal, and ocular features that are reminiscent of the human spEDS-*SLC39A13* phenotype and highlight a role for altered BMP- and TGFβ-signaling in spEDS-*SLC39A13* pathogenesis.

##### Models of Brittle Cornea Syndrome—Defects in PRDM5

Brittle cornea syndrome (BCS) is clinically hallmarked by a severe ocular phenotype with thin and fragile cornea leading to increased risk of spontaneous rupture and often vision loss. Additionally, patients also present with hearing loss and signs of generalized connective tissue fragility such as distal joint hypermobility and mild cutaneous and craniofacial abnormalities. BCS is caused by biallelic defects in either *ZNF469* or *PRDM5*. *ZNF469* encodes the zinc finger protein ZNF469, which is believed to be a (transcriptional) regulator of the synthesis and/or organization of collagen fibrils (Abu et al., 2008) but its function remains elusive. *PRDM5* encodes PR domain zinc finger protein 5 (PRDM5), a transcription factor that was identified as a putative tumor suppressor gene in several cancers but has also been shown to regulate transcription of collagen and other ECM genes (Galli et al., 2012).

Although no animal models have been generated to study BCS pathogenesis, two independent studies investigated the role of PRDM5 in zebrafish larvae as a putative tumor suppressor and during craniofacial development (Meani et al., 2009; Ding et al., 2013). Interestingly, morpholino-based *prdm5* knockdown affects early stages of zebrafish development with craniofacial malformation including cyclopia or smaller eyes and axial mesendodermal defects (jaw, heart, and blood) (Figure 6D; Meani et al., 2009). Craniofacial malformations with neurocranial defects were subsequently confirmed in *prdm5* knockout (*prdm5*^*hi*61*Tg*^) zebrafish larvae (Amsterdam et al., 2004; Ding et al., 2013). These findings hint to the ocular phenotype and mild craniofacial abnormalities seen in BCS patients. Furthermore, *prdm5* knockdown results in impaired morphogenetic movement during gastrulation as a consequence of increased canonical Wnt-signaling (Meani et al., 2009; Ding et al., 2013).

To date, no stable animal models of BCS due to ZNF469 defects have been described.

#### Models of Additional Ehlers–Danlos Syndromes Variants

##### Models of Classical-Like Ehlers-Danlos Syndrome Type 2—Defects in Adipocyte Enhancer-Binding Protein 1

The most recent defects associated with human EDS reside in adipocyte enhancer-binding protein-1 (AEBP1), also known as aortic carboxypeptidase-like protein (ACLP). AEBP1 is expressed in tissues with a high collagen content and is a secreted ECM-associated protein that preferentially binds to fibrillar types I, III, and V collagen and acts as a positive regulator of collagen polymerization *in vitro* and collagen fibrillogenesis *in vivo* (Layne et al., 2001; Blackburn et al., 2018). Biallelic mutations in the *AEBP1* gene, encoding AEBP1, cause an EDS phenotype hallmarked by skin hyperextensibility with atrophic scarring, generalized joint hypermobility, foot deformities and early-onset osteopenia, provisionally referred to as classical-like EDS type 2 (clEDS2) (Blackburn et al., 2018; Syx et al., 2019).

Before *AEBP1* defects were linked to EDS, two groups independently created homozygous *Aebp1* knockout (*Aebp1*^–^*^/^*^–^) mice, which presented some phenotypic discrepancies. While the majority of the first *Aebp1*^–^*^/^*^–^ mouse model died perinatally due to gastroschisis, surviving *Aebp1*^–^*^/^*^–^ mice developed spontaneous skin lesions and present with delayed wound healing, which correlated with reduced dermal fibroblast proliferation (Layne et al., 2001). These wound healing defects are reminiscent of the phenotype of clEDS2 patients, indicating a role for AEBP1 in wound repair. Additional studies in mice suggested role(s) of AEBP1 in fibroblast-to-myofibroblast transition in lung through TGFβ-signaling (Schissel et al., 2009; Tumelty et al., 2014), and activation of the canonical Wnt-signaling pathway through frizzled-8 and LRP6 in hepatic stellate cells (Teratani et al., 2018). Deregulation of these pathways could potentially contribute to the defective wound healing and bone development and homeostasis seen in patients. For the second model, about half of *Aebp1*^–^*^/^*^–^ mice survived and they were smaller, with markedly reduced white adipose tissue and defective proliferation and lower MAPK activity in *Aebp1*^–^*^/^*^–^ pre-adipocytes. Additionally, lactation defects were observed in females and infertility in males (Ro et al., 2007; Zhang et al., 2011).

As such, studies in *Aebp1*^–^*^/^*^–^ mice suggest the involvement of AEBP1 in the development and homeostasis of adipose tissue, mammary gland and connective tissue, with recapitulation of the dermal manifestations of clEDS2 in one model. Nevertheless, the exact mechanisms leading to the observed EDS phenotype remain currently unknown.

### Naturally Occurring Ehlers–Danlos Syndromes Models

Before the first genetically engineered EDS mouse models were generated, naturally occurring EDS(-like) phenotypes were already described for decades in domesticated animals. There are several reports of cats, dogs, rabbits, mink, cattle, sheep, and horses displaying a disease reminiscent of EDS (Fjølstad and Helle, 1974; Hegreberg, 1975; Counts et al., 1977; Holbrook et al., 1980; Brown et al., 1993; Hansen et al., 2015). The phenotypes of these animals were hallmarked by thin and hyperextensible skin, which was fragile leading to hemorrhagic wounds and atrophic scars; and commonly described as cutis hyperelastica, hyperelastosis cutis, dermatosparaxis, dermal/collagen dysplasia, dermal/cutaneous asthenia, or Ehlers–Danlos-like syndromes (Halper, 2013).

While the genetic defects causing EDS-like pathologies in domestic animals were less well studied in early reports, the advent of next generation sequencing technologies such as whole exome sequencing and whole genome sequencing, provided molecular proof of the underlying disorder in more recent reports. Mutations have been reported in *Col5a1* in cats (Spycher et al., 2018) and dogs (Bauer et al., 2019a), *Col5a2* in cattle (Jacinto et al., 2020), *Adamts2* in cattle (Colige et al., 1999), sheep (Zhou et al., 2011; Monteagudo et al., 2015; Joller et al., 2017), and dogs (Jaffey et al., 2019), *Plod1* in horse (Monthoux et al., 2015), *Tnxb* in dogs (Bauer et al., 2019b) and *B4galt7* in Finish horse (Leegwater et al., 2016). These animals display phenotypic features reminiscent of the corresponding human EDS counterpart. Additionally, horses presenting with an EDS-like condition with loose skin have been described and coined Hereditary Equine Regional Dermal Asthenia (HERDA). This condition is caused by defects in the *Ppib* gene, encoding cyclophilin B. This gene is not associated with EDS in humans but results in OI (van Dijk et al., 2009; Rashmir-Raven, 2013). Functional studies in these animals are restricted but sometimes accompanied by biochemical, histopathological and/or ultrastructural data. A summary of the findings of spontaneous models with confirmed genetic defects is provided in Table 2.

**TABLE 2:** Overview of naturally occurring animals with a molecularly proven EDS subtype.

Gene (EDS subtype)	Molecular defect	Species (breed)	Phenotype	References
*Col5a1* (cEDS)	c.3420delG, p.(Leu1141Serfs*134)	Cat [Domestic shorthair *(F)*]	Multiple recurrent skin tears with little or no bleeding, mainly on the dorsal neck and shoulders Skin hyperextensibility Previous lacerations healed slowly and left shiny alopecic scars Bilateral hip subluxation Bilateral carpal hyperextension with plantigrade appearance Pain and laxity during palpation of all joints Bilateral perineal hernias	(Spycher et al., 2018)
*Col5a1* (cEDS)	c.3030delG, p.(Gly1013Valfs*260)	Dog [Labrador *(M)*]	*(4m)*: Clinically healthy Normal skeletal development Small skin lacerations of the distal limbs, hock and tail base Seroma-like swelling in regions of skin trauma (hocks, tail base): seroma or hematoma formation *(6m)*: Generalized joint hypermobility ( >180°) Skin hyperextensibility Skin fragility	(Bauer et al., 2019a)
*Col5a2* (cEDS)	c.2366G>T, p.(Gly789Val)	Cattle [Holstein calf *(3d, F)*]	Hyperextensibility of the neck skin Atrophic scarring Skin fragility Wavy, short, thin collagen in the skin surrounded by edema Moderate to severe acute hemorrhages	(Jacinto et al., 2020)
*Adamts2* (dEDS)	Homozygous deletion of 20bp and insertion of 3bp, p.[(Val153fs*24)];[(Val153fs*24)]	Cattle	Dermatosparaxis phenotype Extreme skin fragility Disorganized collagen bundles	(Colige et al., 1999)
*Adamts2* (dEDS)	c.[424G>T];[424G>T], p.[(Glu142*)];[(Glu142*)]	Sheep [White Dorper lambs *(2d)*]	Dermatosparaxis phenotype Severe skin wounds Skin tears Easily separated skin from subcutaneous tissue	(Zhou et al., 2011; Joller et al., 2017)
*Adamts2* (dEDS)	c.[805G>A];[805G>A], p.[(Val269Met)];[(Val269Met)]	Sheep	Dermatosparaxis phenotype Severe skin wounds	(Monteagudo et al., 2015)
*Adamts2* (dEDS)	c.[769C>T];[769C>T], p.[(Arg257*)];[(Arg257*)]	Dog [Doberman Pinscher *(8w)*]	Hypermobile carpal, tarsal and stifle joints Loose and hyperelastic skin Wounds and large atrophic scars	(Jaffey et al., 2019)
*Plod1* (kEDS)	c.[2032G>A];[2032G>A], p.[Gly678Arg];[Gly678Arg]	Horse [Westfalian Warmblood *(5y, F)*]	Warmblood Fragile Foal Syndrome (WFFS) Skin lesions Incomplete closure of the abdominal wall Hyperflexion/extension of joints Inability to stand Difficulty breathing Often die within 72 h	(Monthoux et al., 2015; Martin et al., 2020)
*Tnxb* (clEDS1)	c.[2012G>A];[2900G>A], p.[(Ser671Asn)];[(Gly967Asp)]	Dog [Mixed-breed *(F)*]	Fragile skin, easily teared or bruised Poor healing wounds Hyperextensible skin *(21m)*	(Bauer et al., 2019b)
*B4galt7* (spEDS)	c.[50G>A];[50G>A], p.[(Arg17Lys)];[(Arg17Lys)]	Horses (Friesian Horses)	Dwarfism Inwards protruding ribs Flexor tendon laxity Hyperextension of the fetlock joints	(Leegwater et al., 2016)
*Ppib* (/)	c.[115G>A];[115G>A] p.[(Gly39Arg)];[(Gly39Arg)]	Horse (American Quarter horse)	Hereditary Equine Regional Dermal Asthenia (HERDA) Hyperextensible, fragile and thin skin Ulcerations and skin degeneration Hematoma	(Tryon et al., 2007)

*Molecular defects are indicated at cDNA and protein level. When available, age (d: days, w: weeks, m: months, y: years) and sex (M: male, F: female) are included between parentheses.*

Taken together, it is clear that EDS also occurs naturally in mammals and can be prominent in specific (in)breeding schemes. However, due to limited resources, especially in sequencing capacity for animals, many cases are not molecularly confirmed, and in-depth functional analyses are often lacking.

## Conclusion and Future Perspectives

For 14 of the 20 hitherto known EDS-associated genes, animal models, either mouse or zebrafish, have been created. While for the more common cEDS and vEDS subtypes different engineered mouse models are available and often extensively characterized, animal models are lacking for some of the rarer EDS subtypes (e.g., periodontal EDS, kEDS-*FKBP14*, arthrochalasia EDS and BCS-*ZNF469*). Recently, zebrafish models were introduced into the EDS research field [*col1a2*^–/–^ (cvEDS) and *b4galt7* and *b3galt6*^–/–^ (spEDS)]. Additionally, numerous domestic animals displaying an EDS(-like) phenotype have been reported, with the genetic defect identified for some. Although these models are valuable tools to learn about the EDS phenotype in animals other than rodents or zebrafish, they are less amendable for large-scale research purposes.

Especially engineered models have been instrumental in discerning the functions of these particular proteins during development, maturation and repair and in portraying their roles during collagen biosynthesis and fibrillogenesis, for some even before their contribution to an EDS phenotype was elucidated. Extensive phenotypical characterization of these models has shown that they largely phenocopy their human counterparts, with recapitulation of several key clinical features of the corresponding EDS subtype, including dermal, cardiovascular, musculoskeletal and ocular alterations. Moreover, these models provide valuable tools to perform in-depth studies of several disease-related aspects (e.g., wound healing); and allow to investigate organs and tissues that are not easily accessible from humans (e.g., cardiovascular or nervous tissue) to assess physicochemical, ultrastructural and biomechanical properties during early development and adult stages. This can be particularly helpful to elucidate disease mechanisms in rarer EDS subtypes for which only a limited number of patients are reported. Since for most EDS types, the exact link between a single gene defect and the observed phenotype remains elusive, more studies are needed to investigate the underlying pathways and understand the pathophysiological consequences.

As such, investigation of these models hinted toward the involvement of several signaling pathways, such as Wnt-signaling in *prdm5* knockdown zebrafish and *Aebp1*^–/–^ mice, BMP1- and/or TGFβ-signaling in *Slc39a13-KO* and *Aebp1*^–/–^ mice; and PLC/IP_3_/PKC/ERK-signaling in *Col3a1* knock-in mice and highlighted potential compensatory mechanisms such as the presence of a trilinker region in *b3galt6*^–/–^ zebrafish. Further validation of these findings in patient samples is warranted and additional investigations are required to further pinpoint common or distinct pathways in several EDS subtypes, thereby explaining phenotypic overlap and differences.

Nevertheless, direct comparison between different EDS models is limited and complicated by several variables, such as: (1) differences in genetic background influencing the phenotypic outcome and survival of animals (e.g., *Chst14*^–/–^ mice) (Montagutelli, 2000); (2) age at which the animals were examined; and (3) anatomical location of investigated tissues. Furthermore, sex differences have been described in most murine models of vEDS, with a more severe skin and vascular phenotype in males compared to females. Although the sex of the EDS animals was not always clearly indicated in literature, male-female divergences are increasingly being recognized in several organ systems, including, skin thickness, cardiovascular function, musculoskeletal integrity as well as in physiological processes, such as pain, and need to be taken into account in future preclinical EDS research (Huxley, 2007; Lang, 2011; Rosen et al., 2017; Sorge and Totsch, 2017; Rahrovan et al., 2018).

The majority of the models generated to date harbor heterozygous or homozygous null mutations, which are indispensable to study the consequences of complete protein deficiency, but which usually do not capture the complete mutation spectrum associated with a particular EDS subtype (e.g., structural variants like glycine substitutions or exon skips in *COL5A1*, *COL5A2*, or *COL12A1*). With exception of *Col3a1* models of vEDS, no knock-in EDS models have been developed. Advances in genome editing technologies such as CRISPR/Cas9 and base editing can assist in creating these models (Adli, 2018; Porto et al., 2020).

Currently, treatment and management options for EDS patients are limited and for none of the EDS subtypes a targeted therapy is available. So far, preclinical therapeutic interventions have only been explored in mouse models of vEDS and contradicting results were obtained depending on the model used. When the elucidation of the pathogenic mechanisms and deregulated pathways progresses, novel druggable targets will likely be identified. Additionally, the introduction of zebrafish into the EDS research field holds promising potential for therapeutic discovery, since these animals are highly amenable for drug/compound screening.

Despite the availability of animal models for several EDS subtypes, the study of these models for understanding the pathophysiology of EDS is still in its infancy but they can supply a tremendous bulk of information to the scientific community. The generation of additional models (both murine and zebrafish), harboring different genetic defects and different types of genetic variants, will further boost our knowledge and might lead us into a new era of disease research and drug discovery. In summary, these EDS animal models proved to be valid models that are and will continue to be valuable resources that, when explored to full extend, can uncover novel insights in pathogenic mechanisms, identify reliable biomarkers and pinpoint relevant cellular processes or signaling pathways that can serve as targets for the development of disease-specific (and/or personalized) therapies.

## Author Contributions

RV and DS wrote the manuscript. RM, A-MM, and FM read and edited the draft of the manuscript. All authors approved the final version of the manuscript.

## Conflict of Interest

The authors declare that the research was conducted in the absence of any commercial or financial relationships that could be construed as a potential conflict of interest.

## Publisher’s Note

All claims expressed in this article are solely those of the authors and do not necessarily represent those of their affiliated organizations, or those of the publisher, the editors and the reviewers. Any product that may be evaluated in this article, or claim that may be made by its manufacturer, is not guaranteed or endorsed by the publisher.
