# Supplementary material for: Animal Models of Ehlers–Danlos Syndromes: Phenotype, Pathogenesis, and Translational Potential
Source: Front Genet. 2021 Oct 12;12:726474. doi: 10.3389/fgene.2021.726474 (PMC8547655; doi:10.3389/fgene.2021.726474)
Supplement: Supplementary file 1 [file Presentation_1.pdf]

Supplementary Material

1 Supplementary Data

1.1 Additional mouse models affecting type V collagen

1.1.1 Tendon- and ligament-specific *Col5a1* knockout mice

To study the role of type V collagen in relation to joint hypermobility in more depth and to overcome the embryonic lethality seen in traditional *Col5a1* knockout mice (*Col5a1*$^{-/-}$) (Wenstrup et al., 2004), a conditional tendon- and ligament-specific *Col5a1* knockout model (*Col5a1*$^{\Delta ten/\Delta ten}$) was created (using the scleraxis promotor). Complete loss of type V collagen in tendon and ligaments leads to a more severe joint phenotype in these *Col5a1*$^{\Delta ten/\Delta ten}$ mice compared to the *Col5a1*$^{+/-}$ model, with a smaller body size due to difficulties with feeding, slow movement, abnormal gait in young mice, joint dislocations in older mice, decreased forelimb grip strength, excessive joint laxity (in knee and ankle joints) and hypermobility as well as early-onset knee osteoarthritis (postnatal day 30). These phenotypic changes were accompanied by ultrastructural alterations including abnormal fiber organization as well as altered fibril structure with increased fibril diameter distribution and decreased fibril numbers and was more severe in the major joint stabilizing ligament, the anterior cruciate ligament (ACL), compared to the flexor digitorum longus (FDL) tendon (Sun et al., 2015). Biomechanical analysis showed a reduced stiffness of the FDL tendon, Achilles tendon (ACH), ACL, and supraspinatus tendon (SST), significantly smaller tendons and a reduced modulus only in ACL and SST of *Col5a1*$^{\Delta ten/\Delta ten}$ mice, thereby indicating differences between the examined tissues and providing evidence that mechanical properties of tendons and ligaments are tissue-dependent (Connizzo et al., 2015; Sun et al., 2015). Subsequently a more detailed study was performed focusing on multiscale responses in SST from *Col5a1*$^{+/-}$, *Col5a1*$^{\Delta ten/\Delta ten}$ and wild-type mice. On a macroscale level, there was a dose dependent effect with the most severely reduced performance observed for *Col5a1*$^{\Delta ten/\Delta ten}$ SST (Connizzo et al., 2016). *Col5a1*$^{+/-}$ and *Col5a1*$^{\Delta ten/\Delta ten}$ tendons showed a quicker response to loading through fiber realignment and fibril stretch. *Col5a1*$^{+/-}$ mice showed increased fibril sliding while *Col5a1*$^{\Delta ten/\Delta ten}$ were unable to perform fibril sliding. Although *Col5a1*$^{\Delta ten/\Delta ten}$ mice faithfully recapitulate the joint phenotype of cEDS and serve as a valuable model to study joint instability and related abnormalities in tendon and ligaments, complete *COL5A1* loss is not observed in cEDS patients. Collectively, the studies in these murine models confirm the importance of type V collagen for tendon structure, integrity and recovery from injury as well as for joint stability and soft connective tissue integrity (Johnston et al., 2017).

1.1.2 Corneal stroma-specific *Col5a1* knockout mice

To further study the role of type V collagen during corneal stromal fibrillogenesis, a conditional corneal stroma-specific *Col5a1* knockout (*Col5a1*$^{\Delta st/\Delta st}$) was generated (using the keratocan promotor). *Col5a1*$^{\Delta st/\Delta st}$ mice showed a more severe corneal phenotype with a corneal stromal opacity associated with reduced stromal thickness. *Col5a1*$^{\Delta st/\Delta st}$ mice displayed a reduced amount of larger and less uniform collagen fibrils in the corneal stroma than wild-type, which was more pronounced in the anterior stroma compared to the posterior stroma. In addition, fibrils were poorly organized with disrupted lamellae architecture (Sun et al., 2011).

1.1.3 Mice overexpressing human *COL5A1*

To study the role of $[\alpha1(V)]_3$ homotrimers in skin development, a transgenic mouse model was created that overexpressed the human pro-α1(V) chain exclusively in the epidermis (under the keratin 14 (K14) promoter) (Bonod-Bidaud et al., 2012). The resulting K14-*COL5A1* mice showed accumulation of thin fibrillar $[\alpha1(V)]_3$ homotrimers underneath the epidermal basement membrane where it plays a bridging role in stabilizing the epidermal-dermal interface by interacting with several extracellular matrix (ECM) molecules (*e.g.,* types IV and VI collagen, laminin-111 and tenascin-X). One-month-old transgenic mice did not present skin hyperextensibility but showed altered skin deformity with slower relaxation of the skin fold after holding, and reduced stiffness and rupture stress compared to wild-type skin. Similar to *Col5a2*$^{pN/pN}$ mice, K14-*COL5A1* mice had more hair follicles, pointing to an unexpected role for the type V collagen homotrimer in hair cycling (Bonod-Bidaud et al., 2012).

1.1.4 Conditional *Col5a2* knockdown mice

To further study the role of the α2(V)-chain in adult tissues and overcome the embryonic lethality associated with constitutive *Col5a2* knockout mice, conditional *Col5a2* mouse models were created (Park et al., 2017). Ubiquitous postnatal knockdown of *Col5a2* (using the ubiquitin C (Ubc) promotor) resulted in *Ubc-Cre;Col5a2*$^{fl/fl}$ mice that failed to gain weight following tamoxifen treatment (approximately 40% weight reduction at adulthood), were shorter and had severe kypholordosis compared to wild-type mice. Subscapular skin has a markedly thinner and more compact dermis, which appeared to be more collagen dense. Although the skin is also more fragile, it lacks the characteristic hyperextensibility seen in cEDS patients and in *Col5a1*$^{+/-}$ and *Col5a2*$^{+/-}$ mice. Consistent with human cEDS, ultrastructural analysis of the subscapular dermis of 15-week-old *Ubc-Cre;Col5a2*$^{fl/fl}$ mice showed decreased fibril density with overall increased diameters and the presence of abnormally large ‘cauliflower’ collagen fibrils. Similar to cEDS patients and *Col5a1*$^{+/-}$ mice, severely reduced *in vivo* wound closure was observed in *Ubc-Cre;Col5a2*$^{fl/fl}$ mice. *Ubc-Cre;Col5a2*$^{fl/fl}$ mice lacked white adipose tissue in the skin with absence of identifiable adipocytes and also had severely reduced abdominal fat depots with miniadipocytes of an abnormally small size, suggesting an important role for the α2(V)-chain in white adipose tissue development and maintenance (Park et al., 2017). Lack of dermal white adipose tissue is not a reported characteristic of cEDS, but interestingly, *Col5a3* knockout mice also show subtle reductions in the thickness of dermal white adipose tissue associated with changes in its function (Huang et al., 2011).

To evaluate the role of type V collagen in the predisposition to aortic aneurysms, mice were created with conditional postnatal knockdown of *Col5a2* in gastrointestinal and vascular smooth muscle cells specifically (using the myosin heavy chain 11 (Myh11) promotor). The resulting *Myh11-Cre;Col5a2*$^{fl/fl}$ mice display larger diameters of elastase-induced abdominal aortic aneurysms (Park et al., 2017).

1.2 Additional mouse model affecting type III collagen

In 1986, a mouse with tight skin in the interscapular region, coined Tsk2/+, was identified in a screening following exposure to the mutagenic agent N-ethyl-N-nitrosourea (ENU). Tsk2/+ mice presented phenotypic features resembling human systemic sclerosis, including tight skin, dysregulated ECM deposition, dermal fibrosis and evidence of an autoimmune response (Christner et al., 1998; Gentiletti et al., 2005). The genetic defect was mapped to chromosome 1 and subsequent molecular investigations identified a point mutation in the *Col3a1* gene, resulting in a heterozygous cysteine-to-serine substitution (p.(Cys33Ser)) in the pro-α1(III)-chain. This substitution is located in the N-propeptide domain of type III procollagen and gives rise to increased *Col3a1* expression and type III collagen accumulation in the dermis. As such the Tsk2/+ phenotype is proposed to result from a gain-of-function in contrast to *COL3A1* mutations that are identified in vascular EDS patients (Long et al., 2015).

1.3 Additional mouse model affecting type I collagen

A number of mutations located within the 85 most N-terminal amino acid residues of the triple helical domain of type I collagen, result in inefficient or delayed N-propeptide processing of type I collagen and are associated with an 'EDS/osteogenesis imperfecta (OI) overlap' phenotype, currently not included in the EDS classification (Cabral et al., 2005; Malfait et al., 2017). An ENU-induced dominant mutagenesis screen for low bone mineral density in mice identified a founder harboring a *Col1a1* splice site mutation leading to a skip of exon 9 corresponding to a predicted in-frame deletion of 18 amino acids in the N-terminal part of the type I collagen triple helix. Heterozygous $Col1a1^{Jrt/+}$ mice were smaller in size and show phenotypic features of OI such as lower bone mineral density and mechanically weak and brittle bones that fractured easily as well as EDS, including reduced tensile strength of the skin, more frayed tail tendon and curvature of the spine in about one third of mice (Chen et al., 2014; Eimar et al., 2016). In addition, $Col1a1^{Jrt/+}$ mice show ultrastructural abnormalities in dentin matrix and mineralization. This study suggested that the mutant α1(I)-chains exerted a dominant negative effect on the biosynthesis of type I collagen with a substantial reduction in the amount of normal protein that was available to form the heterotypic type I collagen fibrils. TEM analysis indicated that collagen fibrils in $Col1a1^{Jrt/+}$ bone, tendon, and dermal fibroblast cultures had smaller diameters compared to those in wild-type tissues (Chen et al., 2014). Pain-related behaviors and functional impairment were investigated in the $Col1a1^{Jrt/+}$ mouse model (Abdelaziz et al., 2015). Behavioral testing revealed that $Col1a1^{Jrt/+}$ mice were more sensitive to mechanical and thermal (hot and cold) stimuli compared to wild-type littermates. $Col1a1^{Jrt/+}$ mice also showed decreased distance travelled in open field glass chamber test, decreased rearing attempts, substantial limping and decreased voluntary running in a home cage running wheel. Studying the peripheral innervation revealed no differences in the upper dermis of the hind paw via PGP9.5 staining, or calcitonin gene related protein (CGRP) immunoreactive sensory neurons in lumbar DRG. Although initially described as an EDS/OI overlap model, subsequent reports often refer to $Col1a1^{Jrt/+}$ mice as an OI model, likely due to the more prominent and severe bone phenotype observed in this model (Roschger et al., 2014; Abdelaziz et al., 2015; Boraschi-Diaz et al., 2017; Baglole et al., 2018).

1.4 Dse knockdown in *Xenopus laevis*

To study the role of DS-epi1 during fetal development, morpholino-based knockdown of *Dse* was established in a *Xenopus laevis* model to model mcEDS and neurocristopathies. Knocking down *Dse* impairs the correct activation of transcription factors involved in the epithelial-mesenchymal transition and reduces the extent of neural crest cell migration (Gouignard et al., 2016). This affects craniofacial development origination from neural crest cells (**Supplementary Figure 1**), which is in line with the developmental role and craniofacial alterations seen in mcEDS-*DSE* patients.

2 Supplementary Figures and Tables

2.1 Supplementary Figure

Supplementary Figure 1. Phenotypic findings in a *Xenopus laevis* model caused by morpholino-based knockdown of *Dse* adapted from (Gouignard et al., 2016). Images were used under the terms of the Creative Commons Attribution License.

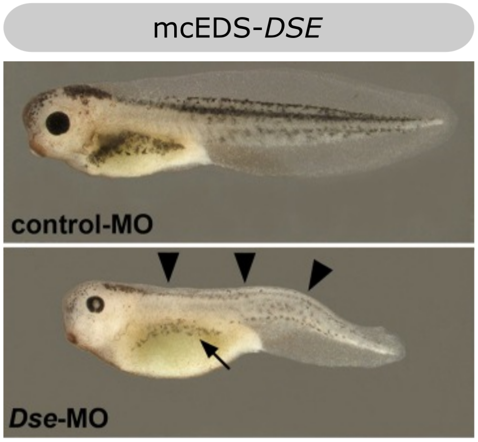

2.2 Supplementary Tables

Supplementary Table 1. Phenotypic characteristics of mouse models with defects in EDS-associated genes. When available, age (dpc: days post conception, d: days, w: weeks, m: months, y: years) and sex (M: male, F: female) are included between parentheses. HI: haploinsufficiency, KO: (homozygous) knockout, KI: knock-in, TEM: transmission electron microscopy, LM: light microscopy, NE: not examined.

Model<br>(EDS type)	Effect, Strategy and Background	General	Skin/<br>Integument	Tendon/<br>Ligament	Cardiovascular	Skeletal	Neuromuscular	Ocular	Other	Ref
Col5a1$^{+/-}$<br>(cEDS)	HI<br><br>heterozygous deletion of majority of exon 3 and 4 of the *Col5a1* gene due to insertion of a neomycin cassette via homologous recombination	- viable<br>- fertile	- thin skin<br>- skin hyperextensibility<br>- decreased tensile strength of normal and wounded skin *(12w)*<br>- delayed dermal collagen accumulation between 4w and 8w, total collagen quantity is normal by 12w<br>- TEM subscapular dermis: 50% reduction in number of collagen fibrils with (1) round, slightly larger collagen fibrils and (2) very large	- reduced tensile strength of flexor digitorum longus tendon *(60d)* and patellar tendon *(120d, M)*<br>- diminished mechanical recovery following bilateral patellar tendon injury *(120d, M)* with smaller collagen fibrils 6w post-injury<br>- TEM flexor digitorum longus: mild abnormalities with less regular cross-sections and smaller diameters of collagen fibrils<br>- TEM patellar tendon: mostly normal circular	- decreased aortic stiffness (descending aorta>ascending aorta) *(12w)*<br>- reduced tensile strength of the aorta (descending aorta>ascending aorta) *(12w)*<br>- heart: histologically normal (*18.5 dpc*)	NE	- mechanical allodynia (*15-20w, M/F)*	- grossly normal cornea without opacity<br>- 26% reduced thickness of the corneal stroma *(12w)*<br>- 14% decrease in collagen content *(5m)*<br>- TEM: increased collagen fibril diameter, 25% reduction in number of fibrils, normal fibril packing	- homozygous *Col5a1*$^{-/-}$ mice are embryonic lethal at 10 dpc due to cardiovascular insufficiency with less blood-filled vessels and pooled blood	(Wenstrup et al., 2004, 2006, 2011; DeNigris et al., 2015; Connizzo et al., 2016; Johnston et al., 2017; Syx et al., 2020)

	C57BL/6<br>J		collagen fibrils with irregular contours *(10d, 6w, 12w, 20w, M)*<br>- spontaneous, non-healing wounds *(6m)*<br>- slower *in vivo* wound healing *(60d)*<br>- delayed *in vitro* wound closure by dermal fibroblasts *(20d)*<br>- abnormal cutaneous innervation *(15w, M/F)*	collagen fibril cross-sections with somewhat increased diameters				(*10d, 6w, 12w)*		
Col5a2$^{pN/pN}$	homozygous in-frame deletion of exon 6 of the *Col5a2* gene leading to severely reduced pro-α2(V) chains<br><br>C57BL/6, 129/Sv/ter	- decreased survival rate (5% past weaning) due to respiratory problems *(48h)*<br><br>Surviving mice:<br>- reduced body weight (50%) *(3w)*	- severe skin fragility (with multiple scars and bleeding lacerations)<br>- increased skin stretchability<br>- histology: reduced dermal and increased hypodermal thickness, unusually localization of hair follicles *(6w)*<br>- TEM: more disorganized, less tightly packed dermal collagen fibrils with heterogeneous sizes *(5d)*	NE	NE	- progressive spinal deformities (lordosis, kyphosis) *(20d)*	NE	- thinner corneal stroma<br>- TEM: disorganization of collagen fibrils with an overall increased diameter *(5d)*	- heterozygous *Col5a2*$^{pN/+}$ mice are grossly normal	(Andrikopoulos et al., 1995; Chanut-Delalande et al., 2004)

			- dermal fibroblasts produce a sparse network of disorganized, thin fibril *in vitro*<br>- increased apoptosis of dermal fibroblasts *in vitro*							
Col5a2$^{+/-}$<br>(cEDS)	HI<br>heterozygous deletion of the promoter region, exon 1 and its enhancer sequences of the *Col5a2* gene using the *LoxP-Cre* system<br>C57BL/6	- viable	- mild skin hyperextensibility<br>- mildly reduced tensile strength<br>- TEM subscapular dermis: mild collagen fibril abnormalities with irregular collagen fibril contours (on longitudinal sections), but lacking 'cauliflowers' *(15w)*	NE	- decreased aortic stiffness (ascending aorta>descending aorta)<br>- reduced tensile strength of the aorta (ascending aorta>descending aorta)	NE	NE	NE	- homozygous *Col5a2*$^{-/-}$ mice are embryonic lethal at 12 dpc due to cardiovascular insufficiency (pallor, congestion of blood and absence of visible vasculature due to paucity of blood in the cardiovascular system)	(Park et al., 2015, 2017)
Col3a1^{tm1Jae}	KO<br>homozygous deletion of the promotor and exon 1 of the *Col3a1*	- 5% survival rate at weaning age (most deaths 48h after birth of unknown cause)<br>- surviving mice:	surviving mice:<br>- spontaneous open skin wounds in 60% of surviving mice (mostly around shoulders)	NE	surviving mice:<br>- rupture of large blood vessels causing death<br>-light microscopy: no alterations in aorta or heart	NE	- brain anomalies (cobblestone-like cortical malformation with pial basement membrane defects and	NE	surviving mice:<br>- intestinal enlargement and occasional rupture causing death<br>- TEM intestine, liver, lung: disorganized and/or reduced numbers of collagen fibrils with	(Liu et al., 1997; Jeong et al., 2012)

	gene via homologous recombination<br><br>BALB/c x C57BL/6	normal appearance,<br>15% smaller, reduced life span of 6 months	- no abnormalities on light microscopy<br>- TEM: disorganized collagen fibrils with highly variable diameters		- TEM: severely reduced or absent collagen fibrils in the aortic media, one third reduction in number and highly variable diameters (twice the size) of collagen fibril in the aortic adventitia, severely reduced or missing collagen fibrils between epicardium and myocardium, underdeveloped microvilli of the heart epicardium		neuronal overmigration)		highly variable diameters	
Col3a1^{tm1Jae}/J<br>(vEDS)	HI<br><br>heterozygous deletion of the promotor and exon 1 of the *Col3a1* gene via homologous recombination<br><br>BALB/c	- phenotypically normal up to 2 years of age<br>- normal body weight<br>- no increased mortality<br>- no craniofacial abnormalities	- subjectively looser skin when handled<br>- increased wound closure *in vitro* by embryonic dermal fibroblasts and *in vivo (>1 y)*<br>- increased scar tissue area formation *in vivo*	NE	- echocardiography: no detectable differences in heart, aorta, or carotid arteries *(9,14,20m, M/F)*<br>- lower aortic stiffness *(10-12m, F)*<br>- histological lesions in the aorta (fragmentation of internal elastic lamina) *(9<21m, M (88%)>F (47%))*	- reduced trabecular bone quantity	NE	NE	- colon: reduced strength and increased compliance, normal histology<br><br>- bladder *(8w M/F)*: increased compliance, decreased neurotransmitter function, TEM: less densely packed collagen fibril with variable diameters.	(Stevenson et al., 2006; Cooper et al., 2010; Briest et al., 2011; Volk et al., 2011, 2014; Jeong et al., 2012; Tae et al., 2012; Faugeroux et al., 2013; Goudot et al., 2018)

Col3a1$^{m1Lsmi/+}$<br><br>(vEDS)	heterozygous in-frame deletion of exon 33-39 of the *Col3a1* gene (NM_009930.2:c.2281_2820del, NP_034060.2:p.(Pro762_Gly941del))<br><br>129Ola x C57BL/6J<br><br>or<br><br>C57BL/6J*	- sudden death in 28% due to acute aortic dissection *(mostly 4-10w, M>F)*	- no overt skin phenotype<br><br>- TEM abdominal skin: less collagen fibrils with variable and larger diameters *(17w)**	NE	- acute aortic dissection in 28% (M:F ratio = 2:1) without prior hypertension or aneurysm *(mostly 4-10w)*<br><br>- elevated blood pressure *(14w M)*<br><br>- reduced maximal tensile force of the thoracic aorta *(4-5w M, 6-7w M/F, 9-11w M/F)*<br><br>- reduced collagen content in the thoracic aortic wall *(11-45w)**<br><br>- TEM thoracic aorta: reduced electron density of the elastic lamellae with variable width and disrupted architecture *(4w)*, less collagen fibrils with variable and larger diameters in adventitia *(17w)**	NE	NE	NE	- no evidence of gastrointestinal complications or rupture<br><br>- homozygous *Col3a1*$^{m1Lsmi/m1Lsmi}$ mice are embryonic lethal (<9.5 dpc)	(Smith et al., 2011),<br><br>(Dubacher et al., 2019)

Col3a1$^{Tg\text{-}G182S}$<br><br>(vEDS)	transgenic<br><br>7-fold overexpression of type III collagen harboring the p.(Gly182Ser) substitution<br><br>C57BL/6J	- viable	- thin and easily torn skin upon handling<br>- spontaneous transdermal wounds requiring euthanasia *(13-14w, M)*<br>- reduced tensile strength of the abdominal skin *(12w)*<br>- reduced dermal collagen content *(12w)*<br>- TEM: malformed and loosely packed collagen fibrils, which were reduced in number in the dermis with highly variable diameters, of which some were extremely large *(12w)*, no signs of dilated endoplasmic reticulum or autophagosomes in dermal fibroblasts *(12w)*	NE	- reduced tensile strength of the aorta *(12w)*<br>- reduced thickness of the adventitia with a lower total collagen content *(12w)*<br>- TEM: collagen fibrils with a variable diameter and irregular contour in the adventitia, *(12w)* abnormal distribution and morphology of smooth muscle cells and reduced contact with the elastic lamina *(12w)*	NE	NE	NE	- no evidence of gastrointestinal complications or rupture	(D'hondt et al., 2018)

Col3a1$^{G183R/+}$<br><br>(vEDS)	KI<br><br>Glycine substitution: p.(Gly183Arg)<br><br>Not indicated	- viable<br><br>- increased mortality rate *(F: 25% and M: 60%, 24w)*<br><br>- no weight difference *(8w)*	NE	NE	- spontaneous thoracic aortic rupture without dilatation with a mortality rate of 25% in female and 60% in male mice *(24w)*<br><br>- no alterations in heartbeat *(8w)*<br><br>- lower aortic stiffness *(3-5m, M/F)*<br><br>- no alterations in elastic fibers in aortas *(24w)*<br><br>- TEM aorta: lower density of collagen fibrils with heterogeneous diameters *(24w)*, dilated endoplasmic reticulum in adventitial fibroblasts *(24w)*	NE	NE	NE	- decreased number of homozygous mice born (not further evaluated)	(Fontaine et al., 2015; Goudot et al., 2018)
Col3a1$^{G209S/+}$<br><br>(vEDS)	KI<br><br>Glycine substitution: Gly209Ser<br><br>CRISPR-Cas9<br><br>C57BL/6J	- mild phenotype<br><br>- mean survival: 400d	NE	NE	- spontaneous aortic rupture<br><br>- TEM: disrupted elastic lamellar units with thickened elastic fibers with a moth-eaten appearance, disarray of vascular smooth muscle cells, paucity of collagen fibrils	NE	NE	NE	NE	(Bowen et al., 2019)

Col3a1$^{G938D/+}$<br><br>(vEDS)	KI<br><br>Glycine substitution: Gly938Asp<br><br>CRISPR-Cas9<br><br>C57BL/6J	- severe phenotype<br><br>- smaller body size<br><br>- mean survival: 45d	NE	NE	- spontaneous aortic rupture<br><br>- smaller aorta *(2m)*<br><br>- reduced collagen content *(2m)*<br><br>- TEM: disrupted elastic lamellar units with thickened elastic fibers with a moth-eaten appearance, disarray of vascular smooth muscle cells, paucity of collagen fibrils with variable, generally smaller, diameters in the aortic media, dilated endoplasmic reticulum of adventitial fibroblasts	NE	NE	NE	NE	(Bowen et al., 2019)
Adamts2$^{-/-}$<br><br>(dEDS)	KO<br><br>replacement of part of intron 13, exon 14 and part of intron 14 of the *Adamts2* gene by a	- viable<br><br>- no differences at birth<br><br>- *2m*: triangular face, shorter snout, less dense hair<br><br>- F: fertile	- thinner and softer skin<br><br>- extremely fragile skin with easy tearing upon handling<br><br>- TEM: unremarkable *(2d)*, unusually curled morphology of	NE	- normal histology and ultrastructure of the aorta *(2m)*: normal aortic wall thickness, normal elastin lamellae, uniform collagen fibril diameters	- skeleton: unremarkable (no growth retardation)	NE	NE	- mild dental changes with normal incisors but subtle loss of the surface contour of the molar teeth<br><br>- lung abnormalities with decreased parenchymal density and a pseudo-	(Li et al., 2001; Goff et al., 2006)

	neomycin-resistance gene by homologous recombination<br><br>129/SvJ x C57BL/6	- M: sterile	collagen fibrils *(2m)*						emphysematous appearance	
Plod1$^{-/-}$<br>(kEDS)	KO<br><br>homologous recombination with in-frame insertion of lacZ-neo cassette into exon 2 and deletion of a genomic region containing exons 3-6 of the *Plod1* gene<br><br>C57BL/6	- viable<br>- increased mortality rate (15%) *(M/F, <1y)*<br>- fertile	- no skin hyperextensibility<br>- no skin fragility<br>- TEM: collagen fibrils with a variable diameter and irregular contour *(1m, M)*	NE	- aortic rupture (15% of mice die before 1y of age, mostly 1-4m) *(M (17%) /F (9%))*<br>- normal aortic wall thickness in surviving mice<br>- TEM aorta: collagen fibrils with a variable diameter and irregular contour *(1m, M)*, less regularly ordered vascular smooth muscle cells with vacuolization and mitochondrial swelling *(1m, M)*	- no kypho-scoliosis	- muscle hypotonia (flaccid and powerless movements)<br>- gait abnormalities	- TEM cornea: normal	NE	(Takaluoma et al., 2007)

Tnxb$^{-/-}$ (clEDS)	KO<br><br>deletion of exon 2 of the *Tnxb* gene by insertion of a PGK-neomycin cassette via homologous recombination<br><br>mixed C57BL/6, CBA, ICR	- viable<br>- morphol-ogically normal at birth<br>- normal growth<br>- fertile	- thicker subcutaneous adipose tissue<br>- TEM: increased collagen fibril diameters with normal fibril density	NE	- decreased blood vessel density with increased diameters in the peripheral nervous system	- reduced femoral bone mass<br>- enhanced osteoclast differentiation and bone-resorbing ability	- increased anxiety-like behavior<br>- superior sensorimotor coordination and emotional learning and memory<br>- mechanical allodynia<br>- increased pain-response to chemical stimulus<br>- reduced diameters of myelinated fibers<br>- TEM sciatic nerve: reduced collagen fibril density in the endoneurium in older, but not young animals	NE	NE	(Matsumoto et al., 2001; Kawakami and Matsumoto, 2011; Hashimoto et al., 2018; Okuda-Ashitaka et al., 2020)
Tnxb$^{-/-}$ (clEDS)	KO<br><br>replacement of exon 1-5 of the *Tnxb* gene by insertion of a LacZ-neomycin cassette via homologo	- viable<br>- morphol-ogically normal at birth<br>- normal growth<br>- fertile	- progressive skin hyperextensibility *(2m)*<br>- TEM: decreased collagen fibril density with normal size and shape<br>- increased dermal elastin density<br>- macroscopically normal *in vivo* wound closure of dorsal skin	- no hypermobility of the tail<br>- no difference in medial collateral ligament stiffness<br>- TEM tail and Achilles tendon: decreased collagen fibril density	NE	NE	- mild muscle weakness	NE	- gastric dysfunction with abnormal gastric sensory function<br>- rectal prolapse *(<1%, F)*	(Mao et al., 2002; Egging et al., 2006, 2007, 2008; Huijing et al., 2010; Voermans et al., 2011)

	us recombination<br><br>mixed C57BL/6, 129/SvJ, FVB									
Col12a1$^{-/-}$<br>(mEDS)	KO<br><br>replacement of exon 2-5 of the *Col12a1* gene with a neomycin cassette via homologous recombination<br><br>C57BL/6 x 129/SvJ	- smaller	NE	- TEM flexor digitorum longus tendon: decreased collagen fibril packing *(30d)*	NE	- shorter, more slender and fragile long bones<br>- aberrant vertebrae structures<br>- kypho-scoliosis	- mild muscle weakness<br>- TEM gastrocnemius muscle: diffusely localized collagen fibrils throughout the endomysium *(30d)*	NE	NE	(Izu et al., 2011, 2016, 2020; Zou et al., 2014)
Chst14$^{-/-}$<br>(mcEDS)	KO<br><br>replacement of the single *Chst14* exon by a neomycin - kanamycin cassette via homologo	- reduced viability (8%)<br><br>surviving mice:<br>- normal life span<br>- smaller	surviving mice:<br>- increased skin fragility	NE	NE	NE	surviving mice:<br>- decreased neurogenesis and diminished neural stem cell proliferation<br>- impaired spatial learning and memory	NE	surviving mice:<br>- abnormally increased tooth growth ("elephant teeth")	(Bian et al., 2011; Akyüz et al., 2013; Rost et al., 2016)

	us recombination<br><br>C57BL/6 x 129/SvJ	- reduced fertility<br>- kinked tail								
Chst14-/-<br>*(Chst14^{tm1Lex})*<br>(mcEDS)	KO<br><br>replacement of the single *Chst14* exon<br><br>C57BL/6 x 129/SvJ<br>or<br>BALB/c*	- perinatal lethality (1.3% survival in adulthood)	- reduced tensile strength *(F)**<br>- TEM: decreased collagen fibril density with increased intrafibrillar spaces, and disorganized collagen fibers*	NE	- placental vascular abnormalities with smaller vascular diameters in placental villi (part of the fetal placenta)	NE	NE	NE	- placentas: reduced weight, hypoxia- and/or necrotic-like changes (in 6%)	(Tang et al., 2010; Hirose et al., 2020)
Dse$^{-/-}$<br>(mcEDS)	KO<br><br>insertion of neomycin cassette in exon 2 of the *Dse* gene<br><br>C57BL/6 x 129/SvJ<br>or<br>NFR*	- smaller (5-10%)<br>- kinked tail (resolved before 4w)<br>- reduced fertility<br>- normal body weight*<br>- kinked tail*	- reduced tensile strength<br>- LM: sparser loose hypodermal connective tissue<br>- TEM: dermal collagen fibrils with larger diameters	- TEM tail tendon: mostly normal collagen fibrils with a minor shift toward thicker fibril diameters<br>- TEM Achilles tendon: normal	NE	NE	- neural tube defects (e.g., exencephaly and spina bifida) (5%)*	NE	- abdominal wall defect with herniated intestines (16%)*	(Maccarana et al., 2009; Gustafsson et al., 2014; Stachtea et al., 2015)

Slc39a13-KO (spEDS)	KO<br><br>genomic deletion of exons 6–8 of the *Slc39a13* gene by insertion of a neomycin cassette<br><br>C57BL/6 *	- viable<br>- growth retardation *(M/F)*<br>- cranio-facial abnormalities: sunken and downslanting eyes	-thin and fragile skin<br>- thinner subcutaneous adipose tissue<br>- TEM: smaller and less densely packed collagen fibrils *(5w)*	NE	- no aneurysms or arterial ruptures<br>- histology and TEM of the thoracic aorta: abnormalities in the tunica media (potentially resulting in increased fragility) *(12w)**	-progressive kyphosis *(3-4w)*<br>- osteopenia<br>- abnormal cartilage development	NE	- reduced corneal stroma thickness<br>-TEM: smaller and more widely spaced collagen fibrils	- dental abnormalities: incisor deformities and aberrant dentin formation *(5w)*<br><br>- heterozygous mice are phenotypically normal	(Fukada et al., 2008; Hirose et al., 2015, 2018)
Aebp1$^{-/-}$ (clEDS2)	KO<br><br>replacement of exons 7-16 of the *Aebp1* gene with a neomycin cassette via homologous recombination<br><br>129/SvJ x C57BL/6	- perinatally lethality due to gastroschisis (>90%)	surviving *Aebp1*$^{-/-}$ mice (6.6%):<br>- spontaneous skin lesions<br>- delayed wound healing	NE	NE	NE	NE	NE	NE	(Layne et al., 2001).

Aebp1$^{-/-}$ (clEDS2)	KO<br><br>replacement of exon 7-12 of the *Aebp1* gene with a neomycin cassette via homologous recombination<br><br>C57BL/6	- 50% survival rate<br>- normal growth rate (*<5w, M/F*)<br>- decreased body weight *(20w, M (-22%)/F (-24%))*<br>- *M*: infertile	NE	NE	NE	NE	NE	NE	- *F*: lactation inability due to defective secretory activation<br>- Reduction in white adipose tissue *(7-9m, M>F)*	(Ro et al., 2007; Zhang et al., 2011)

Supplementary Table 2. Phenotypic characteristics of zebrafish models with defects in EDS-associated genes. When available, age (hpf: hours post fertilization, dpf: days post fertilization, m: months) and the model used are included between parentheses. BCS: brittle cornea syndrome, KO: knockout, HM: hypomorphic, KD: (morpholino-based) knockdown, SBMO: splice-blocking morpholino, TBMO: translation-blocking morpholino, PTC: premature termination codon, NE: not examined.

**Model** (EDS type)	**Effect and strategy**	**General**	**Skin/ Integument**	**Tendon/ Ligament**	**Cardiovascular**	**Skeletal**	**Neuromuscular**	**Ocular**	**Other**	**Ref**
col1a2$^{-/-}$ (cvEDS) (sa17981, zebrafish international resource center)	KO homozygous *col1a2* splice site mutation resulting in a frameshift (p.(Ala154Cysfs*23) and complete absence of the pro-α2(I)-chain	- viable	- interruption of the typical stripe pattern - increased skin fragility - reduced dermal thickness (50%) - decreased strength of a skin flap (soft connective tissue) *(5m)*	- local distortion and dislocation of the intervertebral ligament with normal elastin layer, but reduced notochord sheath layer and complete loss of type I collagen fibrous ligament	- normal histology of cardiac valves - normal cardiac function and blood flow	- kyphosis at the level of transitioning precaudal to caudal vertebrae (more mechanical stress) - local alterations in bone thickness - slightly lower mineralization in the vertebral column - fusions at some of the vertebral endplates - loss of typical Sharpey fibers - no rib fracture calluses	NE	NE	- heterozygous *col1a2*$^{+/-}$ zebrafish are asymptomatic	(Gistelinck et al., 2018)

b4galt7 *'morphant'* (spEDS)	KD<br><br>Splice-blocking morpholino (SBMO) introducing partial retention of exon 3 resulting in a PTC<br><br>or<br><br>Translation-blocking morpholino (TBMO)	For both SBMO and TBMO *(4dpf)*:<br><br>- small, round head<br><br>- compres-sion and underdevelopment of the lower jaw<br><br>- small, bent pectoral fins<br><br>- smaller (attributed to small head)	NE	NE	NE	- severely reduced or absent mineralized bone structures *(4dpf)*<br><br>- delayed intramembranous and endochondral ossification *(4dpf)*<br><br>- delayed notochord sheath mineralization *(4dpf)*<br><br>- less developed, misshapen and partly absent cartilage structures in the lower jaw<br><br>- Meckel's cartilage and ceratohyal are distorted, and the five pharyngeal arches are missing.<br><br>- disturbed chondrocyte stacking in the ceratohyal *(5 dpf)*	- decreased hatching *(2dpf, TBMO only)*<br><br>- touch-evoked escape response *(2dpf, TMBO only)*<br><br>- disturbed filamentous actin patterning in trunk muscle *(3dpf, TMBO only)*<br><br>- no difference in type I collagen in trunk muscle	NE	- reduced amounts of HS and CS GAGs *(TMBO > SBMO)*<br><br>- Decreased and more diffuse type I collagen staining in regions with reduced cartilage formation and disrupted chondrocyte intercalation	(Delbaere et al., 2019)

b4galt7^{sgRNA1}<br><br>*b4galt7^{sgRNA2}*<br><br>*'crispant'*<br><br>(spEDS)	HM<br><br>CRISPR/Cas9 (mosaic F0 generation, *crispant*)	- small, round head *(4dpf)*<br><br>- compres-sed jaw *(4dpf)*<br><br>- small, bent pectoral fins *(4dpf)*<br><br>- smaller (attributed to small head) *(4dpf)*<br><br>- more variable phenotypic appearance (depending on indel frequency)	NE	NE	NE	- severely reduced/absent mineralized bone structures *(4dpf)*<br><br>- delayed intramembranous and endo-chondral ossifi-cation *(4dpf)*<br><br>- delayed notochord sheath mineral-ization *(4dpf)*<br><br>- less abundant and more disorganized cranial neural crest (CNC) cells in pharyngeal arches<br><br>- disturbed chondrocyte stacking in the ceratohyal *(5dpf)*<br><br>- cartilage structures in the lower jaw are less developed, misshapen and partly absent<br><br>- Meckel's cartilage and ceratohyal are distorted, and the five pharyngeal arches are missing	- normal hatching *(2dpf, sgRNA1)*<br><br>- normal touch-evoked escape response *(2dpf, sgRNA1)*<br><br>- normal filamentous actin patterning in trunk muscle *(3dpf, sgRNA1)*	NE	- reduced amounts of HS and CS GAGs	(Delbaere et al., 2019)

b4galt7$^{-/-}$<br>(spEDS)	KO<br>CRISPR/Cas9 (>F1 generation)<br>KO1: c.122_125del (cmg45)<br>KO2: c.129_142del (cmg47)	- head and pectoral fin malformations *(4dpf)*<br>- lethal at 10 dpf	NE	NE	NE	- delayed or absent intramembranous and endochondral ossification of several bone structures *(5 dpf)*<br>- complete lack of cartilage staining *(4dpf)*<br>- disorganized chondrocyte stacking in the head *(4dpf)*	- normal filamentous actin patterning in trunk muscle *(3dpf, KO1)*	NE	- complete loss of HS and CS GAGs	(Delbaere et al., 2019)
b3galt6$^{-/-}$<br>(spEDS)	KO<br>CRISPR/Cas9 (>F1 generation)<br>KO1: c.181delinsAAAGAGCTCCTAAAGGG, p.(Phe68∗) (cmg20)<br>KO2: c.398_401del, p.(Asn13	- viable<br>- reduced body length *(4m)*<br>- de-formed, small and round head	- interruptions of the blue horizontal stripes<br>- TEM: thicker epidermis covering the scales, loosely packed dermal collagen fibrils with increased interfibrillar spaces *(5m)*	- TEM intervertebral ligament: less organized type I collagen fibrils *(5m)*	NE	- progressive craniofacial and skeletal abnormalities *(20dpf, 4m)*<br>- kyphosis and scoliosis *(20dpf, 4m)*<br>- extra intramembranous bone and bony elements on the vertebrae *(4m)*<br>- generalized reduction in bone volume and thickness and relatively increased tissue mineral density *(4m)*	- reduced critical swimming speed and endurance *(8m)*<br>- thicker endomysium around dorsolateral muscle fibers *(5m)*<br>- TEM dorsolateral muscle: increased sarcomere length *(5m)*	NE	- decreased HS, CS and DS concentration in bone, skin and muscle<br>- presence of non-canonical trilinker region	(Delbaere et al., 2020)

	9*) (cmg22)					- TEM: disturbed collagen fibril organization in vertebral bone with electron dense spots *(5m)*				
prdm5 (BCS)	KD<br><br>ATG blocking morpholino<br>or<br>splice blocking morpholino (SBMO)	- cranio-facial malformation with axial mesendodermal defects (jaw, heart, blood) *(SBMO only, 48hpf)*	NE	NE	NE	NE	NE	- cyclopia *(ATG blocking morpholino, 48hpf)*<br>- small eyes *(SBMO, 48hpf)*	- impaired morphogenetic movements (*e.g.*, reduced distance between eye/telencephalon and the mid-hindbrain border) due to over-activation canonical Wnt-signaling *(SBMO)*	(Meani et al., 2009)
prdm5^{hi61Tg} (BCS)	KO<br><br>Viral insertion in the first exon, predicted to be a complete null	- slightly rounder head *(5dpf)*<br>- cranio-facial malformation with shortened and narrower neurocranium *(5dpf)*	NE	NE	NE	NE	NE	NE	- slightly underdeveloped liver/gut *(5dpf)*	(Amsterdam et al., 2004; Ding et al., 2013)

Supplementary Table 3. Overview of the preclinical studies and pharmacological interventions in mouse models of vEDS. MMP: matrix metalloproteinases.

**Model (start of treatment)**	**Compound *(mechanism of action)***	**Dose, administration and regimen**	**Readout**	**Ref**
Col3a1^{tm1Jae}/J (6-month-old females)	Doxycyclin *(broad-spectrum antibiotic and MMP inhibitor)*	25 mg/kg/day in food for 3 months	Reduced MMP activity in carotids, prevented stress-induced aortic pathology	(Briest et al., 2011)
Col3a1^{tm1Jae}/J (6-month-old females)	Doxycyclin *(broad-spectrum antibiotic and MMP inhibitor)*	100 mg/kg/day in food for 3 months	Reduced MMP activity in carotids, attenuated the decreased aortic collagen content and prevented stress-induced aortic pathology	(Briest et al., 2011)
Col3a1^{tm1Jae}/J (3 weeks old)	Doxycyclin *(broad-spectrum antibiotic and MMP inhibitor)*	100 mg/kg/day in food for 9 months	Reduced MMP activity, normalized the reduced aortic collagen content and prevention of spontaneous aortic lesions	(Tae et al., 2012)
Col3a1$^{m1Lsmi/+}$ (4 weeks old)	Celiprolol *(β1 receptor antagonist and partial β2 receptor agonist)*	200 mg/kg/day in drinking water for 4 weeks	Increased maximal tensile force of the thoracic aorta	(Dubacher et al., 2019)
Col3a1$^{m1Lsmi/+}$ (4 weeks old)	Doxycyclin *(broad-spectrum antibiotic and MMP inhibitor)*	100 mg/kg/day in food for 4 weeks	Increased maximal tensile force of the thoracic aorta	(Dubacher et al., 2019)
Col3a1$^{m1Lsmi/+}$ (4 weeks old)	Losartan *(angiotensin II receptor type 1 antagonist)*	180 mg/kg/day in drinking water for 4 or 8 weeks	No change in maximal tensile force of the thoracic aorta	(Dubacher et al., 2019)
Col3a1$^{m1Lsmi/+}$ (4 weeks old)	Bisoprolol *(selective β receptor antagonist)*	100 mg/kg/day in drinking water for 4 weeks	No change in maximal tensile force of the thoracic aorta	(Gorosabel et al., 2019)

Col3a1$^{G938D/+}$ (weaning, P21)	Losartan *(angiotensin II receptor type 1 antagonist)*	60 mg/kg/day in drinking water until 2 months of age	No effect on survival	(Bowen et al., 2019)
Col3a1$^{G938D/+}$ (weaning, P21)	Propranolol *(non-selective β receptor antagonist)*	80 mg/kg/day in drinking water until 2 months of age	No effect on survival	(Bowen et al., 2019)
Col3a1$^{G938D/+}$ (weaning, P21)	Atenolol *(selective β1 receptor antagonist)*	120 mg/kg/day in drinking water until 2 months of age	No effect on survival	(Bowen et al., 2019)
Col3a1$^{G938D/+}$ (weaning, P21)	Amlodipine besylate *(calcium antagonist)*	12 mg/kg/day in drinking water until 2 months of age	No effect on survival (but trend towards increased risk of aortic rupture)	(Bowen et al., 2019)
Col3a1$^{G938D/+}$ (weaning, P21)	Celiprolol *(β1 receptor antagonist and partial β2 receptor agonist)*	200 mg/kg/day in drinking water for 45 days	Accelerated death	(Bowen et al., 2019)
Col3a1$^{G209S/+}$ (P60)	Celiprolol *(β1 receptor antagonist and partial β2 receptor agonist)*	200 mg/kg/day in drinking water for 45 days	Accelerated death	(Bowen et al., 2019)
Col3a1$^{G938D/+}$ (weaning, P21)	Ruboxistaurin *(PKCβ inhibitor)*	8 mg/kg/day mixed with powdered food for 45 days	94% survival compared to 52% without treatment	(Bowen et al., 2019)
Col3a1$^{G938D/+}$ (weaning, P21)	Cobimetinib *(MEK inhibitor)*	2 mg/kg/day in drinking water for 45 days	90% survival compared to 52% without treatment	(Bowen et al., 2019)
Col3a1$^{G938D/+}$ (at birth)	Hydralazine *(IP_3/PKC inhibitor)*	32 mg/kg/day in drinking water for 100 days	97% survival till 45 days but lost at puberty (around 50 days) with survival of 25% of males and 60% of females	(Bowen et al., 2019)
Col3a1$^{G938D/+}$ (at birth + weaning, P21)	Hydralazine *(IP_3/PKC inhibitor)* + Bicalutamide *(androgen*	32 mg/kg/day in drinking water for 100 days + 50 mg/kg/day mixed with powdered food for	90% survival in male mice and 100% survival in female mice	(Bowen et al., 2019)

	receptor antagonist)	2 months (males) or 80 days (females)		
Col3a1$^{G938D/+}$ (weaning, P21)	Bicalutamide *(androgen receptor antagonist)*	50 mg/kg/day mixed with powdered food for 60 days	70% survival after 60 days of treatment versus 50% survival in untreated mice	(Bowen et al., 2019)
Col3a1$^{G938D/+}$ (at birth)	Hydralazine *(IP$_3$/PKC inhibitor)* + Spironolactone *(competitive antagonist of the androgen receptor)*	32 mg/kg/day in drinking water for 65 days + 100 mg/kg/day mixed with powdered food for 65 days	100% survival in both sexes versus 43% survival in untreated animals	(Bowen et al., 2019)
Col3a1$^{G209S/+}$ (third week of pregnancy)	Oxytocin receptor antagonist	1 µg/kg/hr via continuous infusion subcutaneously by a mini Alzet pump for 5 weeks	90% postpartum survival versus 46% in untreated females	(Bowen et al., 2019)
Col3a1$^{G209S/+}$ (third week of pregnancy)	Trametinib *(MEK inhibitor)*	1 mg/kg/day by oral gavage for 5 weeks	95% postpartum survival versus 46% in untreated females	(Bowen et al., 2019)
Col3a1$^{G209S/+}$ (third week of pregnancy)	Hydralazine *(IP$_3$/PKC inhibitor)*	16 mg/kg/day in drinking water for 5 weeks	95% postpartum survival versus 46% in untreated females	(Bowen et al., 2019)
Col3a1$^{G209S/+}$ (third week of pregnancy)	Propranolol (systemic blood pressure reduction)	80 mg/kg/day in drinking water for 5 weeks	No impact on postpartum survival	(Bowen et al., 2019)

Supplementary Table 4. Overview of SLRP deficient mouse models mimicking features of EDS. When available, age (E: embryonic day, w: weeks, m: months, y: years) and sex (M: male, F: female) are included between parentheses. KO: (homozygous) knockout, TEM: transmission electron microscopy, LM: light microscopy, NE: not examined.

Model	Effect and strategy	General	Skin/Integument	Tendon/Ligament	Cardiovascular	Musculoskeletal	Ocular	Other	Ref
Dcn$^{-/-}$<br>(Decorin)	KO<br>disruption of exon 2 of the *Dcn* gene via homologous recombination<br>129Sv x B1/Swiss	- viable<br>- normal size<br>- fertile	- skin laxity and fragility *(3m)*<br>- sharp detachment of tail skin between the deeper dermis and the fascia<br>- TEM: collagen less orderly packed, variable shape and size compared to WT littermates	- very irregular, ragged outline of fibril cross-sections in tail tendon<br>- tail tendon: no change in mechanical properties<br>- patellar tendons: increased modulus and stress relaxation	NE	- no bone abnormalities	- no significant changes in the cornea	- hypomineralized dentin and delayed enamel formation<br>- decreased dentin sialoprotein, dentin matrix protein-1, bone sialoprotein and osteopontin<br>- no difference in in the levels of albumin, globulin, electrolytes, sodium, and chloride, in the hepatic enzymes, ALT and AST, the number of white and red blood cells, hematocrit and hemoglobin	(Danielson et al., 1997; Goldberg et al., 2005; Robinson et al., 2005)
Lum$^{tm1Sc/tm1Sc}$<br>(Lumican)	KO<br>deletion of exon 2 of the *Lum* gene via homologous recombination<br>129/Sv+^{Tyr}+^{p}	- Mendelian rate of birth<br>- viable<br>- 10-15% of offspring were smaller at birth	- loose and fragile skin<br>- increased skin compliance<br>- reduced tensile strength *(9-10m, M)*<br>- LM: disorganized and more loosely arranged dermal connective tissue, poorly aligned fibroblasts<br>- TEM dorsal skin: larger and abnormally	NE	- no major cardiac valvular anomalies	NE	- corneal stroma: largely unaffected *(E13.5-E16.5)*<br>- bilateral corneal clouding, with a peripheral ring-like clear zone, increase with age *(5-34w)*<br>- TEM: thicker and irregular shaped collagen fibrils, with more interfibrillar	- intestinal histology: normal	(Chakravarti et al., 1998)

			shaped collagen fibrils				spacing, disorganized keratocytes<br>- normal decorin levels (whole eyes)		
Dpt$^{-/-}$<br>(Dermatopontin)	KO<br>interruption of exon 1 of the *Dpt* gene via homologous recombination<br>129/Sv x C57BL/6	- Mendelian rates of birth<br>- viable<br>- normal size<br>- fertile	- increased skin elasticity<br>- LM dorsal dermis: reduced thickness and collagen content<br>- TEM dermis: collagen fibrils with great variety in diameter and irregular contours *(F)*	- LM: tail tendon showed no differences	- LM: heart looked normal	NE	- LM cornea: reduced stromal thickness *(2 m)*<br>- TEM cornea: increased fibril spacing within the posterior lamellae *(F)*	- liver, lung and kidney: no differences<br>- decreased soluble collagen content	(Takeda et al., 2002; Cooper et al., 2006)
Mimecan$^{-/-}$	KO<br>interruption of exon 2 om the mimecan gene via homologous recombination<br>129/O1a x C57BL/6	- viable<br>- fertile	- moderately reduced tensile strength<br>- TEM dorsal and tail skin: thicker, and less orderly packed collagen fibrils in the skin with variable size and altered morphology	NE	NE	NE	- no significant changes in the cornea *(2-14m)*<br>- TEM: thicker collagen fibrils and less orderly packed	NE	(Tasheva et al., 2002)

3 References

Abdelaziz, D. M., Abdullah, S., Magnussen, C., Ribeiro-da-Silva, A., Komarova, S. V., Rauch, F., et al. (2015). Behavioral signs of pain and functional impairment in a mouse model of osteogenesis imperfecta. *Bone* 81, 400–406. doi:10.1016/j.bone.2015.08.001.

Akyüz, N., Rost, S., Mehanna, A., Bian, S., Loers, G., Oezen, I., et al. (2013). Dermatan 4-O-sulfotransferase1 ablation accelerates peripheral nerve regeneration. *Exp Neurol* 247, 517–530. doi:10.1016/j.expneurol.2013.01.025.

Amsterdam, A., Nissen, R. M., Sun, Z., Swindell, E. C., Farrington, S., and Hopkins, N. (2004). Identification of 315 genes essential for early zebrafish development. *P Natl Acad Sci Usa* 101, 12792–12797. doi:10.1073/pnas.0403929101.

Andrikopoulos, K., Liu, X., Keene, D. R., Jaenisch, R., and Ramirez, F. (1995). Targeted mutation in the col5a2 gene reveals a regulatory role for type V collagen during matrix assembly. *Nat Genet* 9, 31–36. doi:10.1038/ng0195-31.

Baglole, C. J., Liang, F., Traboulsi, H., Souza, A. R. de, Giordano, C., Tauer, J. T., et al. (2018). Pulmonary and diaphragmatic pathology in collagen type I α1 mutant mice with osteogenesis imperfecta. *Pediatr Res* 83, 1165–1171. doi:10.1038/pr.2018.36.

Bian, S., Akyüz, N., Bernreuther, C., Loers, G., Laczynska, E., Jakovcevski, I., et al. (2011). Dermatan sulfotransferase Chst14/D4st1, but not chondroitin sulfotransferase Chst11/C4st1, regulates proliferation and neurogenesis of neural progenitor cells. *J Cell Sci* 124, 4051–4063. doi:10.1242/jcs.088120.

Bonod-Bidaud, C., Roulet, M., Hansen, U., Elsheikh, A., Malbouyres, M., Ricard-Blum, S., et al. (2012). In Vivo Evidence for a Bridging Role of a Collagen V Subtype at the Epidermis–Dermis Interface. *J Invest Dermatol* 132, 1841–1849. doi:10.1038/jid.2012.56.

Boraschi-Diaz, I., Wang, J., Mort, J. S., and Komarova, S. V. (2017). Collagen Type I as a Ligand for Receptor-Mediated Signaling. *Aip Conf Proc* 5, 12. doi:10.3389/fphy.2017.00012.

Bowen, C. J., Giadrosic, J. F. C., Burger, Z., Rykiel, G., Davis, E. C., Helmers, M. R., et al. (2019). Targetable cellular signaling events mediate vascular pathology in vascular Ehlers-Danlos syndrome. *J Clin Invest*. doi:10.1172/jci130730.

Briest, W., Cooper, T. K., Tae, H.-J., Krawczyk, M., McDonnell, N. B., and Talan, M. I. (2011). Doxycycline Ameliorates the Susceptibility to Aortic Lesions in a Mouse Model for the Vascular Type of Ehlers-Danlos Syndrome. *J Pharmacol Exp Ther* 337, 621–627. doi:10.1124/jpet.110.177782.

Cabral, W. A., Makareeva, E., Colige, A., Letocha, A. D., Ty, J. M., Yeowell, H. N., et al. (2005). Mutations Near Amino End of α1(I) Collagen Cause Combined Osteogenesis Imperfecta/Ehlers-Danlos Syndrome by Interference with N-propeptide Processing. *J Biol Chem* 280, 19259–19269. doi:10.1074/jbc.m414698200.

Chakravarti, S., Magnuson, T., Lass, J. H., Jepsen, K. J., LaMantia, C., and Carroll, H. (1998). Lumican Regulates Collagen Fibril Assembly: Skin Fragility and Corneal Opacity in the Absence of Lumican. *J Cell Biology* 141, 1277–1286. doi:10.1083/jcb.141.5.1277.

Chanut-Delalande, H., Bonod-Bidaud, C., Cogne, S., Malbouyres, M., Ramirez, F., Fichard, A., et al. (2004). Development of a Functional Skin Matrix Requires Deposition of Collagen V Heterotrimers. *Mol Cell Biol* 24, 6049–6057. doi:10.1128/mcb.24.13.6049-6057.2004.

Chen, F., Guo, R., Itoh, S., Moreno, L., Rosenthal, E., Zappitelli, T., et al. (2014). First Mouse Model for Combined Osteogenesis Imperfecta and Ehlers-Danlos Syndrome. *J Bone Miner Res* 29, 1412–1423. doi:10.1002/jbmr.2177.

Christner, P. J., Hitraya, E. G., Peters, J., McGrath, R., and Jiménez, S. A. (1998). Transcriptional activation of the α1(I) procollagen gene and up-regulation of α1(I) and α1(III) procollagen messenger RNA in dermal fibroblasts from tight skin 2 mice. *Arthritis Rheumatism* 41, 2132–2142. doi:10.1002/1529-0131(199812)41:12<2132::aid-art8>3.0.co;2-w.

Connizzo, B. K., Freedman, B. R., Fried, J. H., Sun, M., Birk, D. E., and Soslowsky, L. J. (2015). Regulatory role of collagen V in establishing mechanical properties of tendons and ligaments is tissue dependent. *J Orthopaed Res* 33, 882–888. doi:10.1002/jor.22893.

Connizzo, B. K., Han, L., Birk, D. E., and Soslowsky, L. J. (2016). Collagen V-heterozygous and -null supraspinatus tendons exhibit altered dynamic mechanical behaviour at multiple hierarchical scales. *Interface Focus* 6, 20150043. doi:10.1098/rsfs.2015.0043.

Cooper, L. J., Bentley, A. J., Nieduszynski, I. A., Talabani, S., Thomson, A., Utani, A., et al. (2006). The Role of Dermatopontin in the Stromal Organization of the Cornea. *Invest Ophth Vis Sci* 47, 3303–3310. doi:10.1167/iovs.05-1426.

Cooper, T. K., Zhong, Q., Krawczyk, M., Tae, H.-J., Müller, G. A., Schubert, R., et al. (2010). The Haploinsufficient Col3a1 Mouse as a Model for Vascular Ehlers-Danlos Syndrome. *Vet Pathol* 47, 1028–1039. doi:10.1177/0300985810374842.

Danielson, K. G., Baribault, H., Holmes, D. F., Graham, H., Kadler, K. E., and Iozzo, R. V. (1997). Targeted Disruption of Decorin Leads to Abnormal Collagen Fibril Morphology and Skin Fragility. *J Cell Biology* 136, 729–743. doi:10.1083/jcb.136.3.729.

Delbaere, S., Clercq, A. D., Mizumoto, S., Noborn, F., Bek, J. W., Alluyn, L., et al. (2020). b3galt6 knock-out zebrafish recapitulate β3GalT6-deficiency disorders in human and reveal a trisaccharide proteoglycan linkage region. *Front. Cell Dev. Biol.* 8:597857. doi: 10.3389/fcell.2020.597857

Delbaere, S., Damme, T. V., Syx, D., Symoens, S., Coucke, P., Willaert, A., et al. (2019). Hypomorphic zebrafish models mimic the musculoskeletal phenotype of β4GalT7-deficient Ehlers-Danlos syndrome. *Matrix Biol*. doi:10.1016/j.matbio.2019.12.002.

DeNigris, J., Yao, Q., Birk, E. K., and Birk, D. E. (2015). Altered dermal fibroblast behavior in a collagen V haploinsufficient murine model of classic Ehlers–Danlos syndrome. *Connect Tissue Res* 57, 1–9. doi:10.3109/03008207.2015.1081901.

D'hondt, S., Guillemyn, B., Syx, D., Symoens, S., Rycke, R. D., Vanhoutte, L., et al. (2018). Type III collagen affects dermal and vascular collagen fibrillogenesis and tissue integrity in a mutant Col3a1 transgenic mouse model. *Matrix Biol* 70, 72–83. doi:10.1016/j.matbio.2018.03.008.

Ding, H., Clouthier, D. E., and Artinger, K. B. (2013). Redundant roles of PRDM family members in zebrafish craniofacial development. *Dev Dynam* 242, 67–79. doi:10.1002/dvdy.23895.

Dubacher, N., Münger, J., Gorosabel, M. C., Crabb, J., Ksiazek, A. A., Caspar, S. M., et al. (2019). Celiprolol but not losartan improves the biomechanical integrity of the aorta in a mouse model of vascular Ehlers–Danlos syndrome. *Cardiovasc Res*. doi:10.1093/cvr/cvz095.

Egging, D. F., Vlijmen, I. van, Starcher, B., Gijsen, Y., Zweers, M. C., Blankevoort, L., et al. (2006). Dermal connective tissue development in mice: an essential role for tenascin-X. *Cell Tissue Res* 323, 465–474. doi:10.1007/s00441-005-0100-5.

Egging, D. F., Vlijmen-Willems, I. van, Choi, J., Peeters, A. C. T. M., Rens, D. van, Veit, G., et al. (2008). Analysis of obstetric complications and uterine connective tissue in tenascin-X-deficient humans and mice. *Cell Tissue Res* 332, 523–532. doi:10.1007/s00441-008-0591-y.

Egging, D., Vlijmen-Willems, I. van, Tongeren, T. van, Schalkwijk, J., and Peeters, A. (2007). Wound Healing in Tenascin-X Deficient Mice Suggests that Tenascin-X is Involved in Matrix Maturation Rather than Matrix Deposition. *Connect Tissue Res* 48, 93–98. doi:10.1080/03008200601166160.

Eimar, H., Tamimi, F., Retrouvey, J.-M., Rauch, F., Aubin, J. E., and McKee, M. D. (2016). Craniofacial and Dental Defects in the Col1a1 Jrt/+ Mouse Model of Osteogenesis Imperfecta. *J Dent Res* 95, 761–768. doi:10.1177/0022034516637045.

Faugeroux, J., Nematalla, H., Li, W., Clement, M., Robidel, E., Frank, M., et al. (2013). Angiotensin II promotes thoracic aortic dissections and ruptures in Col3a1 haploinsufficient mice. *Hypertens Dallas Tex 1979* 62, 203–8. doi:10.1161/hypertensionaha.111.00974.

Fontaine, E., Faugeroux, J., Beugnon, C., Verpont, M.-C., Nematalla, H., Bruneval, P., et al. (2015). Caractérisation d'un modèle murin du Syndrome d'Ehlers-Danlos vasculaire. *J Mal Vascul* 40, 119. doi:10.1016/j.jmv.2014.12.024.

Fukada, T., Civic, N., Furuichi, T., Shimoda, S., Mishima, K., Higashiyama, H., et al. (2008). The Zinc Transporter SLC39A13/ZIP13 Is Required for Connective Tissue Development; Its Involvement in BMP/TGF-β Signaling Pathways. *Plos One* 3, e3642. doi:10.1371/journal.pone.0003642.

Gentiletti, J., McCloskey, L. J., Artlett, C. M., Peters, J., Jimenez, S. A., and Christner, P. J. (2005). Demonstration of Autoimmunity in the Tight Skin-2 Mouse: A Model for Scleroderma. *J Immunol* 175, 2418–2426. doi:10.4049/jimmunol.175.4.2418.

Gistelinck, C., Kwon, R. Y., Malfait, F., Symoens, S., Harris, M. P., Henke, K., et al. (2018). Zebrafish type I collagen mutants faithfully recapitulate human type I collagenopathies. *Proc National Acad Sci* 115, 201722200. doi:10.1073/pnas.1722200115.

Goff, C. L., Somerville, R. P. T., Kesteloot, F., Powell, K., Birk, D. E., Colige, A. C., et al. (2006). Regulation of procollagen amino-propeptide processing during mouse embryogenesis by specialization of homologous ADAMTS proteases: insights on collagen biosynthesis and dermatosparaxis. *Development* 133, 1587–1596. doi:10.1242/dev.02308.

Goldberg, M., Septier, D., Rapoport, O., Iozzo, R. V., Young, M. F., and Ameye, L. G. (2005). Targeted Disruption of Two Small Leucine-rich Proteoglycans, Biglycan and Decorin, Excerpts Divergent Effects on Enamel and Dentin Formation. *Calcified Tissue Int* 77, 297–310. doi:10.1007/s00223-005-0026-7.

Gorosabel, M. C., Dubacher, N., Meienberg, J., and Matyas, G. (2019). Vascular Ehlers-Danlos syndrome: Can the beneficial effect of celiprolol be extrapolated to bisoprolol? *European Hear J - Cardiovasc Pharmacother* 6, 199–200. doi:10.1093/ehjcvp/pvz067.

Goudot, G., Papadacci, C., Dizier, B., Baudrie, V., Ferreira, I., Boisson-Vidal, C., et al. (2018). Arterial Stiffening with Ultrafast Ultrasound Imaging Gives New Insight into Arterial Phenotype of Vascular Ehlers-Danlos Mouse Models. *Ultraschall Der Medizin - European J Ultrasound* 40, 734–742. doi:10.1055/a-0599-0841.

Gouignard, N., Maccarana, M., Strate, I., Stedingk, K. von, Malmström, A., and Pera, E. M. (2016). Musculocontractural Ehlers–Danlos syndrome and neurocristopathies: dermatan sulfate is required for Xenopus neural crest cells to migrate and adhere to fibronectin. *Dis Model Mech* 9, 607–620. doi:10.1242/dmm.024661.

Gustafsson, R., Stachtea, X., Maccarana, M., Grottling, E., Eklund, E., Malmström, A., et al. (2014). Dermatan sulfate epimerase 1 deficient mice as a model for human abdominal wall defects. *Birth Defects Res Part Clin Mol Teratol* 100, 712–720. doi:10.1002/bdra.23300.

Hashimoto, K., Kajitani, N., Miyamoto, Y., and Matsumoto, K. (2018). Wound healing-related properties detected in an experimental model with a collagen gel contraction assay are affected in the absence of tenascin-X. *Exp Cell Res* 363, 102–113. doi:10.1016/j.yexcr.2017.12.025.

Hirose, T., Mizumoto, S., Hashimoto, A., Takahashi, Y., Yoshizawa, T., Nitahara-Kasahara, Y., et al. (2020). Systematic investigation of the skin in Chst14−/− mice: a model for skin fragility in musculocontractural Ehlers–Danlos syndrome caused by CHST14 variants (mcEDS-CHST14). *Glycobiology*. doi:10.1093/glycob/cwaa058.

Hirose, T., Ogura, T., Tanaka, K., Minaguchi, J., Yamauchi, T., Fukada, T., et al. (2015). Comparative study of dermal components and plasma TGF-β1 levels in Slc39a13/Zip13-KO mice. *J Vet Med Sci* 77, 1385–1389. doi:10.1292/jvms.15-0015.

Hirose, T., Suzuki, I., Takahashi, N., Fukada, T., Tangkawattana, P., and Takehana, K. (2018). Morphometric analysis of cornea in the Slc39a13/Zip13-knockout mice. *J Vet Med Sci* 80, 18–0019. doi:10.1292/jvms.18-0019.

Huang, G., Ge, G., Wang, D., Gopalakrishnan, B., Butz, D. H., Colman, R. J., et al. (2011). α3(V) collagen is critical for glucose homeostasis in mice due to effects in pancreatic islets and peripheral tissues. *J Clin Investigation* 121, 769–83. doi:10.1172/jci45096.

Huijing, P. A., Voermans, N. C., Baan, G. C., Busé, T. E., Engelen, B. G. M. van, and Haan, A. de (2010). Muscle characteristics and altered myofascial force transmission in tenascin-X-deficient mice, a mouse model of Ehlers-Danlos syndrome. *J Appl Physiol* 109, 986–995. doi:10.1152/japplphysiol.00723.2009.

Izu, Y., Adams, S. M., Connizzo, B. K., Beason, D. P., Soslowsky, L. J., Koch, M., et al. (2020). Collagen XII mediated cellular and extracellular mechanisms regulate establishment of tendon structure and function. *Matrix Biol*. doi:10.1016/j.matbio.2020.10.004.

Izu, Y., Ezura, Y., Koch, M., Birk, D. E., and Noda, M. (2016). Collagens VI and XII form complexes mediating osteoblast interactions during osteogenesis. *Cell Tissue Res* 364, 623–635. doi:10.1007/s00441-015-2345-y.

Izu, Y., Sun, M., Zwolanek, D., Veit, G., Williams, V., Cha, B., et al. (2011). Type XII collagen regulates osteoblast polarity and communication during bone formation. *J Cell Biology* 193, 1115–30. doi:10.1083/jcb.201010010.

Jeong, S.-J., Li, S., Luo, R., Strokes, N., and Piao, X. (2012). Loss of Col3a1, the Gene for Ehlers-Danlos Syndrome Type IV, Results in Neocortical Dyslamination. *Plos One* 7, e29767. doi:10.1371/journal.pone.0029767.

Johnston, J. M., Connizzo, B. K., Shetye, S. S., Robinson, K. A., Huegel, J., Rodriguez, A. B., et al. (2017). Collagen V haploinsufficiency in a murine model of classic Ehlers–Danlos syndrome is associated with deficient structural and mechanical healing in tendons. *J Orthopaed Res* 35, 2707–2715. doi:10.1002/jor.23571.

Kawakami, K., and Matsumoto, K. (2011). Behavioral Alterations in Mice Lacking the Gene for Tenascin-X. *Biological Pharm Bulletin* 34, 590–593. doi:10.1248/bpb.34.590.

Layne, M. D., Yet, S.-F., Maemura, K., Hsieh, C.-M., Bernfield, M., Perrella, M. A., et al. (2001). Impaired Abdominal Wall Development and Deficient Wound Healing in Mice Lacking Aortic Carboxypeptidase-Like Protein. *Mol Cell Biol* 21, 5256–5261. doi:10.1128/mcb.21.15.5256-5261.2001.

Li, S.-W., Arita, M., Fertala, A., Bao, Y., Kopen, G. C., Långsjö, T. K., et al. (2001). Transgenic mice with inactive alleles for procollagen N-proteinase (ADAMTS-2) develop fragile skin and male sterility. *Biochem J* 355, 271. doi:10.1042/0264-6021:3550271.

Liu, X., Wu, H., Byrne, M., Krane, S., and Jaenisch, R. (1997). Type III collagen is crucial for collagen I fibrillogenesis and for normal cardiovascular development. *Proc National Acad Sci* 94, 1852–1856. doi:10.1073/pnas.94.5.1852.

Long, K. B., Li, Z., Burgwin, C. M., Choe, S. G., Martyanov, V., Sassi-Gaha, S., et al. (2015). The Tsk2/+ Mouse Fibrotic Phenotype Is Due to a Gain-of-Function Mutation in the PIIINP Segment of the Col3a1 Gene. *J Invest Dermatol* 135, 718–727. doi:10.1038/jid.2014.455.

Maccarana, M., Kalamajski, S., Kongsgaard, M., Magnusson, S. P., Oldberg, A., and Malmström, A. (2009). Dermatan sulfate epimerase 1-deficient mice have reduced content and changed distribution of iduronic acids in dermatan sulfate and an altered collagen structure in skin. *Mol Cell Biol* 29, 5517–28. doi:10.1128/mcb.00430-09.

Malfait, F., Francomano, C., Byers, P., Belmont, J., Berglund, B., Black, J., et al. (2017). The 2017 international classification of the Ehlers–Danlos syndromes. *Am J Medical Genetics Part C Seminars Medical Genetics* 175, 8–26. doi:10.1002/ajmg.c.31552.

Mao, J. R., Taylor, G., Dean, W. B., Wagner, D. R., Afzal, V., Lotz, J. C., et al. (2002). Tenascin-X deficiency mimics Ehlers-Danlos syndrome in mice through alteration of collagen deposition. *Nat Genet* 30, 421–425. doi:10.1038/ng850.

Matsumoto, K., Takayama, N., Ohnishi, J., Ohnishi, E., Shirayoshi, Y., Nakatsuji, N., et al. (2001). Tumour invasion and metastasis are promoted in mice deficient in tenascin-X. *Genes Cells* 6, 1101–1111. doi:10.1046/j.1365-2443.2001.00482.x.

Meani, N., Pezzimenti, F., Deflorian, G., Mione, M., and Alcalay, M. (2009). The Tumor Suppressor PRDM5 Regulates Wnt Signaling at Early Stages of Zebrafish Development. *Plos One* 4, e4273. doi:10.1371/journal.pone.0004273.

Okuda-Ashitaka, E., Kakuchi, Y., Kakumoto, H., Yamanishi, S., Kamada, H., Yoshidu, T., et al. (2020). Mechanical allodynia in mice with tenascin-X deficiency associated with Ehlers-Danlos syndrome. *Sci Rep-uk* 10, 6569. doi:10.1038/s41598-020-63499-2.

Park, A. C., Phan, N., Massoudi, D., Liu, Z., Kernien, J. F., Adams, S. M., et al. (2017). Deficits in Col5a2 Expression Result in Novel Skin and Adipose Abnormalities and Predisposition to Aortic Aneurysms and Dissections. *Am J Pathology* 187, 2300–2311. doi:10.1016/j.ajpath.2017.06.006.

Park, A. C., Phillips, C. L., Pfeiffer, F. M., Roenneburg, D. A., Kernien, J. F., Adams, S. M., et al. (2015). Homozygosity and Heterozygosity for Null Col5a2 Alleles Produce Embryonic Lethality and a Novel Classic Ehlers-Danlos Syndrome–Related Phenotype. *Am J Pathology* 185, 2000–2011. doi:10.1016/j.ajpath.2015.03.022.

Ro, H., Zhang, L., Majdalawieh, A., Kim, S., Wu, X., Lyons, P. J., et al. (2007). Adipocyte Enhancer-Binding Protein 1 Modulates Adiposity and Energy Homeostasis. *Obesity* 15, 288–302. doi:10.1038/oby.2007.569.

Robinson, P. S., Huang, T.-F., Kazam, E., Iozzo, R. V., Birk, D. E., and Soslowsky, L. J. (2005). Influence of Decorin and Biglycan on Mechanical Properties of Multiple Tendons in Knockout Mice. *J Biomechanical Eng* 127, 181–185. doi:10.1115/1.1835363.

Roschger, A., Roschger, P., Keplingter, P., Klaushofer, K., Abdullah, S., Kneissel, M., et al. (2014). Effect of sclerostin antibody treatment in a mouse model of severe osteogenesis imperfecta. *Bone* 66, 182–188. doi:10.1016/j.bone.2014.06.015.

Rost, S., Akyüz, N., Martinovic, T., Huckhagel, T., Jakovcevski, I., and Schachner, M. (2016). Germline ablation of dermatan-4O-sulfotransferase1 reduces regeneration after mouse spinal cord injury. *Neuroscience* 312, 74–85. doi:10.1016/j.neuroscience.2015.11.013.

Smith, L. B., Hadoke, P. W. F., Dyer, E., Denvir, M. A., Brownstein, D., Miller, E., et al. (2011). Haploinsufficiency of the murine Col3a1 locus causes aortic dissection: a novel model of the vascular type of Ehlers–Danlos syndrome. *Cardiovasc Res* 90, 182–190. doi:10.1093/cvr/cvq356.

Stachtea, X. N., Tykesson, E., Kuppevelt, T. H. van, Feinstein, R., Malmström, A., Reijmers, R. M., et al. (2015). Dermatan Sulfate-Free Mice Display Embryological Defects and Are Neonatal Lethal Despite Normal Lymphoid and Non-Lymphoid Organogenesis. *Plos One* 10, e0140279. doi:10.1371/journal.pone.0140279.

Stevenson, K., Kucich, U., Whitbeck, C., Levin, R. M., and Howard, P. S. (2006). Functional changes in bladder tissue from type III collagen-deficient mice. *Mol Cell Biochem* 283, 107–114. doi:10.1007/s11010-006-2388-1.

Sun, M., Chen, S., Adams, S. M., Florer, J. B., Liu, H., Kao, W. W.-Y., et al. (2011). Collagen V is a dominant regulator of collagen fibrillogenesis: dysfunctional regulation of structure and function in a corneal-stroma-specific Col5a1-null mouse model. *J Cell Sci* 124, 4096–4105. doi:10.1242/jcs.091363.

Sun, M., Connizzo, B. K., Adams, S. M., Freedman, B. R., Wenstrup, R. J., Soslowsky, L. J., et al. (2015). Targeted Deletion of Collagen V in Tendons and Ligaments Results in a Classic Ehlers-Danlos Syndrome Joint Phenotype. *Am J Pathology* 185, 1436–1447. doi:10.1016/j.ajpath.2015.01.031.

Syx, D., Miller, R. E., Obeidat, A. M., Tran, P. B., Vroman, R., Malfait, Z., et al. (2020). Pain-related behaviors and abnormal cutaneous innervation in a murine model of classical Ehlers-Danlos syndrome. *Pain* 161, 2274–2283. doi:10.1097/j.pain.0000000000001935.

Tae, H.-J., Marshall, S., Zhang, J., Wang, M., Briest, W., and Talan, M. I. (2012). Chronic Treatment with a Broad-Spectrum Metalloproteinase Inhibitor, Doxycycline, Prevents the Development of Spontaneous Aortic Lesions in a Mouse Model of Vascular Ehlers-Danlos Syndrome. *J Pharmacol Exp Ther* 343, 246–251. doi:10.1124/jpet.112.197020.

Takaluoma, K., Hyry, M., Lantto, J., Sormunen, R., Bank, R. A., Kivirikko, K. I., et al. (2007). Tissue-specific Changes in the Hydroxylysine Content and Cross-links of Collagens and Alterations in Fibril Morphology in Lysyl Hydroxylase 1 Knock-out Mice. *J Biol Chem* 282, 6588–6596. doi:10.1074/jbc.m608830200.

Takeda, U., Utani, A., Wu, J., Shinkai, H., Adachi, E., Koseki, H., et al. (2002). Targeted Disruption of Dermatopontin Causes Abnormal Collagen Fibrillogenesis. *J Invest Dermatol* 119, 678–683. doi:10.1046/j.1523-1747.2002.01863.x.

Tang, T., Li, L., Tang, J., Li, Y., Lin, W. Y., Martin, F., et al. (2010). A mouse knockout library for secreted and transmembrane proteins. *Nat Biotechnol* 28, 749–55. doi:10.1038/nbt.1644.

Tasheva, E. S., Koester, A., Paulsen, A. Q., Garrett, A. S., Boyle, D. L., Davidson, H. J., et al. (2002). Mimecan/osteoglycin-deficient mice have collagen fibril abnormalities. *Mol Vis* 8, 407–15.

Voermans, N. C., Verrijp, K., Eshuis, L., Balemans, M. C. M., Egging, D., Sterrenburg, E., et al. (2011). Mild Muscular Features in Tenascin-X Knockout Mice, A Model of Ehlers–Danlos Syndrome. *Connect Tissue Res* 52, 422–432. doi:10.3109/03008207.2010.551616.

Volk, S. W., Shah, S. R., Cohen, A. J., Wang, Y., Brisson, B. K., Vogel, L. K., et al. (2014). Type III Collagen Regulates Osteoblastogenesis and the Quantity of Trabecular Bone. *Calcified Tissue Int* 94, 621–631. doi:10.1007/s00223-014-9843-x.

Volk, S. W., Wang, Y., Mauldin, E. A., Liechty, K. W., and Adams, S. L. (2011). Diminished Type III Collagen Promotes Myofibroblast Differentiation and Increases Scar Deposition in Cutaneous Wound Healing. *Cells Tissues Organs* 194, 25–37. doi:10.1159/000322399.

Wenstrup, R. J., Florer, J. B., Davidson, J. M., Phillips, C. L., Pfeiffer, B. J., Menezes, D. W., et al. (2006). Murine model of the Ehlers-Danlos Syndrome col5a1 haploinsufficiency disrupts collagen fibril assembly at multiple stages. *J Biol Chem* 281, 12888–12895. doi:10.1074/jbc.m511528200.

Wenstrup, R. J., Florer, J., Brunskill, E., Bell, S., Chervoneva, I., and Birk, D. (2004). Type V Collagen Controls the Initiation of Collagen Fibril Assembly. *J Biol Chem* 279, 53331–53337. doi:10.1074/jbc.m409622200.

Wenstrup, R. J., Smith, S. M., Florer, J. B., Zhang, G., Beason, D. P., Seegmiller, R. E., et al. (2011). Regulation of Collagen Fibril Nucleation and Initial Fibril Assembly Involves Coordinate Interactions with Collagens V and XI in Developing Tendon. *J Biol Chem* 286, 20455–20465. doi:10.1074/jbc.m111.223693.

Zhang, L., Reidy, S. P., Bogachev, O., Hall, B. K., Majdalawieh, A., and Ro, H.-S. (2011). Lactation Defect with Impaired Secretory Activation in AEBP1-Null Mice. *Plos One* 6, e27795. doi:10.1371/journal.pone.0027795.

Zou, Y., Zwolanek, D., Izu, Y., Gandhy, S., Schreiber, G., Brockmann, K., et al. (2014). Recessive and dominant mutations in COL12A1 cause a novel EDS/myopathy overlap syndrome in humans and mice. *Hum Mol Genet* 23, 2339–2352. doi:10.1093/hmg/ddt627.
